# Application of divided convective-dispersive transport model to simulate variability of conservative transport processes inside a planted horizontal subsurface flow constructed wetland

**DOI:** 10.1007/s11356-020-10965-z

**Published:** 2020-11-27

**Authors:** Ernő Dittrich, Mihály Klincsik, Dávid Somfai, Anita Dolgos-Kovács, Tibor Kiss, Anett Szekeres

**Affiliations:** 1 Faculty of Engineering and Informatics, Department of Environmental Engineering, University of Pécs, Boszorkány u. 2, Pécs, H-7624 Hungary; 2 Faculty of Engineering and Informatics, Department of Mathematical Sciences, University of Pécs, Boszorkány u. 2, Pécs, H-7624 Hungary; 3 Hidro-consulting Ltd., Budai Nagy Antal u. 1, Pécs, H-7624 Hungary

**Keywords:** Divided convective-dispersive transport (D-CDT) model, Fréchet distribution, Inverse Gaussian distribution, Subsurface flow constructed wetlands, Transport processes, Tracer test, Hydraulic variability inside the constructed wetland

## Abstract

This paper offers a novel application of our model worked out in Maple environment to help understand the very complex transport processes in horizontal subsurface flow constructed wetland with coarse gravel (HSFCW-C). We made tracer measurements: Inside a constructed wetland, we had 9 sample points, and samples were taken from each point at two depths. Our model is a divided convective-dispersive transport (D-CDT) model which makes a fitted response curve from the sum of two separate CDT curves showing the contributions of the main and side streams. Analytical solutions of CDT curves are inverse Gaussian distribution functions. This model was fitted onto inner points of the measurements to demonstrate that the model gives better fitting to the inner points than the commonly used convective-dispersive transport model. The importance of this new application of the model is that it can resemble transport processes in these constructed wetlands more precisely than the regularly used convective-dispersive transport (CDT) model. The model allows for calculations of velocity and dispersion coefficients. The results showed that this model gave differences of 4–99% (of velocity) and 2–474% (of dispersion coefficient) compared with the CDT model and values were closer to actual hydraulic behavior. The results also demonstrated the main flow path in the system.

## Introduction

Constructed wetlands (CWs)—also known as treatment wetlands—are engineered systems for wastewater treatment. Constructed wetlands have a very low or zero energy demand; therefore, operation and maintenance costs are significantly reduced compared to conventional treatment systems (Almuktar et al. 2018).

There are two main types of constructed wetland: free-surface flow systems (FSF-CW) and subsurface flow systems (SSF-CW). SSF-CWs can be further divided according to the direction of the wastewater flow. Wastewater in SSF-CWs runs either horizontally (in HSSF-CWs) or vertically (in VSSF-CWs) towards the filter media. In VSFCWs, there is an unsaturated, non-permanent flow, and in HFSFCWs there is a saturated, non-permanent flow (Wu et al. 2015; Valipour and Ahn 2016). Our experiments and calculations were performed on HFSFCWs only. We investigated HFSFCWs using coarse gravel as filter medium (HFSCW-C). Constructed wetlands can treat a wide variety of polluted water, including municipal, domestic, agricultural, or industrial wastewaters (Vymazal 2009).

There are important differences between the ideal and the actual flow. One of the reasons is weather conditions, such as rainfall (Kadlec 1997, 1999; Rash and Liehr 1999), evapotranspiration (Galvão et al. 2010; Beebe et al. 2014), and snow melting that can have a huge impact on the flow within constructed wetlands. Another important factor is the construction of the CW: the differences in porosity and hydraulic conductivity of filter media in volume and over time (Dittrich and Klincsik 2015a; Licciardello et al. 2019), the active volume of the porous system (Goebes and Younger 2004), and the inlet and outlet positions (Alcocer et al. 2012; Wang et al. 2014; Okhravi et al. 2017). Finally, there are the clogging processes caused by solids accumulation (Carballeira et al. 2016, Lancheros et al. 2017,Liu et al. 2019), biofilm development (Button et al. 2015; Aiello et al. 2016; Vymazal 2018; de Matos et al. 2018), and root density and distribution (De Paoli and Sperling 2013,Tang et al. 2017) .

Due to the factors mentioned above, the hydrodynamic modeling of SFCWs is a challenging task for experts. In these constructions, biofilm activity and root density can be very intense, and more importantly, biofilm development and root system growth over time may also be significantly more rapid (Samsó and Garcia 2013; Rajabzadeh et al. 2015). These processes can affect the micro-porous system, hydraulic conductivity, and clogging processes as well (Tanner and Sukias 1995). It is quite challenging and often problematic to estimate these processes or, even further, to incorporate these factors into a model.

Conservative tracer tests are commonly used to analyze the hydraulic behavior of constructed wetlands (Levenspiel 1972). Scientists have frequently analyzed SFCWs with conservative tracer tests used as experimental tools to gain more detailed information about the internal hydrodynamics of constructed wetlands (Netter 1994; Suliman et al. 2006; Barbagallo et al. 2011; Wang et al. 2014). Our method was also based on tracer tests. Conservative tracer tests allow for calculations of the hydraulic retention time (HRT) and dispersion coefficient (*D*) of a hydraulic system. Some scientists have also conducted the same tests in HSFCWs with the same goal.

Netter (1994) measured two horizontal subsurface flow constructed wetlands. Tracer tests were taken from each CW. They were filled with different, homogeneously mixed media, gravelly sand and sandy gravel, and both filter materials contained fractions of clay and slit. Samples were taken inside the HSFCW and at the effluent point as well. He concluded that the hydraulic behavior varied considerably within the system. A disadvantageous length to width ratio may have caused this problem as this system was characterized mainly by plug flow with little longitudinal dispersion. The tracer test results showed the main flow paths. The two sides of the CWs were the preferred transport paths in the influent region; in the effluent region, the main flow path was at the middle of the CW. The results presented that both soil filters resulted in heterogeneous flow.

Muñoz et al. (2006) performed four tracer studies with bromide as the tracer. They measured four horizontal subsurface flow constructed wetlands. Their results showed that the theoretical retention time was higher in each tracer study than the mean retention time.

Tang et al. (2017) studied the hydraulic performance of HSSFCWs. Hydraulic behavior was determined by tracer tests; the tracer was bromide ion. They performed measurements on four CWs. The porosity was between 41 and 44%. The results showed that the theoretical retention times were higher in all tracer tests than the mean retention time. Birkigt et al. (2017) investigated the flow and transport processes on a pilot-scale horizontal subsurface constructed wetland with tracer tests (bromide, deuterium oxide, and uranine). There was one sampling point inside the CW; samples were taken at three depths. The results showed that the preferred flow along the bottom layer was with 65–70% of mass flowing along the bottom and 14–18% and 16–17% of mass at the middle and top levels. In this case, the actual residence time was lower than the theoretical time.

Richter et al. (2003) measured HSFCW and were the first in the literature who found that the actual HRT could be higher than the theoretical HRT; however, they didn’t find the reason for this phenomenon.

Bonner et al. (2017) completed a tracer test on laboratory-scale HSFCWs with red fluorescent dye used as tracer. There were 13 sampling points in the CW. The results indicated that the actual residence time was bigger than the theoretical one and that there was some back mixing within the system. The porosity was 43%. We had the same findings based on our transport model (Dittrich and Klincsik 2015a). In this paper we intended to prove that this principle is valid with regard to the inner points of HSSFCWs.

Liu et al. (2018) investigated the effect of solids accumulation and root growth on the hydrodynamics of the HSFCWs. They used three laboratory-scale HSFCWs. The tracer was fluorescein sodium. The samples were taken at two points and at three different substrate depths. Their results indicated that the presence of plant root restricted the water flow within the top layer which resulted in the preferential bottom flow phenomenon. The results showed dispersion numbers between 0,09 and 0,16; these numbers were within the acceptable range.

Batchelor and Loots (1996) attempted to fit completely stirred series tank reactor (CSTR) and convection-dispersion transport (CDT) models to their tracer test results which yielded bad fitting results, the reason of which the authors could not exactly clarify. Chazarenc et al. (2003) investigated with fitting CSTR and CDT models as well. Their results showed good fittings with CSTR models 9 out of 10 times. Nonetheless, important parameters, for example, porosity and hydraulic conductivity, were estimated values only. King et al. (1997) conducted a conservative tracer analysis of a gravel-filled HSFCW. They fitted CSTR and CDT models as well; bad fittings were found. Hydrus-2D uses CSTR and CDT models also at the transport module of the software (Langergraber and Simunek 2011; Langergraber et al. 2009; Toscano et al. 2009); results published, nonetheless, indicate that the module needs further improvement.

We have developed a new transport model with better fitting properties than the CSTR and CDT models (Dittrich and Klincsik 2015b). This model is a very effective method to obtain more detailed results about the hydraulic behavior of SFCWs. In the present article, our objective is to show that our model can provide an adequate description of complex transport processes inside HSFCW-Cs.

## Materials and methods

The tracer measurements were made at a HSFCW-C in Hódmezővásárhely, Hungary. The treatment plant treats 1–1.5 m^3^/day of wastewater from a milk room. The main elements of the technology include a septic tank, the pump system, VHSFW, HSFCW-C, and a polishing pond in the sequence listed.

Scientists have used different tracers: in two studies NaBr (Netter 1994; Tanner and Sukias 1995), in one of the cases tritium (Netter 1994), in another case a special fluorescent substance (eriochrome acid red) (Breen and Chick 1995) and in four cases LiCl (Schierup et al. 1990; Netter 1994; King et al. 1997; Rash and Liehr 1999). We chose LiCl as conservative tracer. The absorption capacity of the filter media for LiCl was tested in the Environmental Technological Laboratory of University of Pécs. The findings indicated that LiCl as a conservative tracer is applicable in the examined construction. For further details about the treatment plant and the tracer tests, consult Dittrich and Klincsik (2015a).

**Fig. 1:**
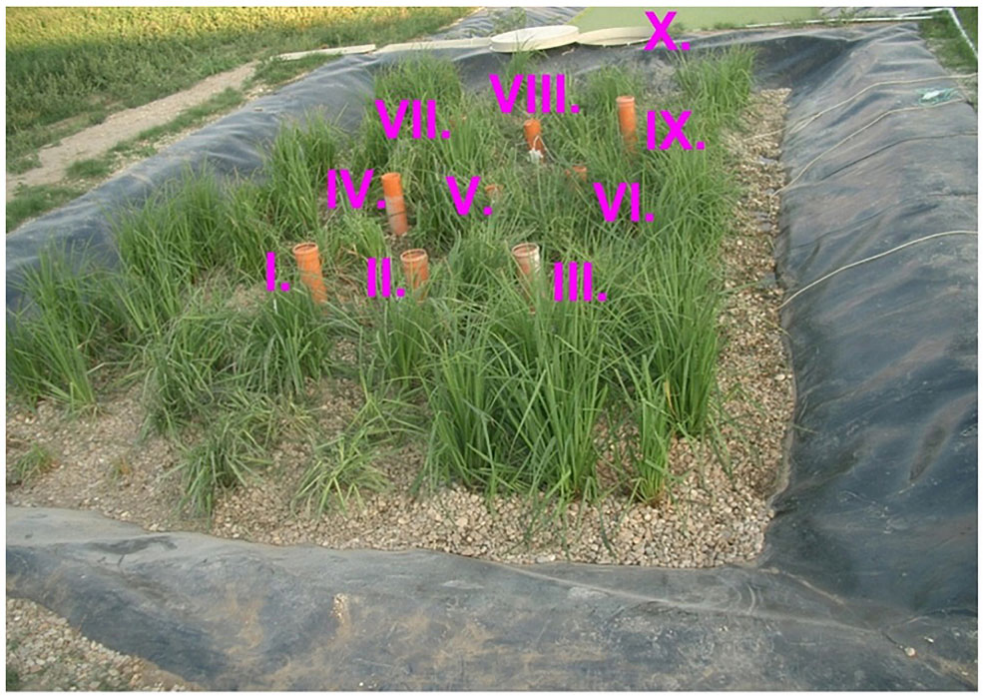
Measurement points in the HSFCW-C in Hódmezővásárhely, Hungary

Inside the CW, there were 9 sample points, and the samples were collected at the effluent. These points are demonstrated in Fig. 1. UNICAM Solaar M atomic absorption device was used for the measurement of the LiCl concentration values.

The measured C-t value pairs and other essential measured parameters are summarized in Appendix 1 (Tables 8, 9, 10, and 11). Four different measurements were performed in time. The measurements obtained S/1, S/2, S/3, and S/4 reference numbers for easier documentation as shown below. The main data of our own tracer measurements are summarized in Appendix 1 (Table 7).

Our aim was to design a more accurate process than the conventional CDT and CSTR models for simulating transport processes in HSFCW-Cs.

We developed an accurate process with the aim to fit the Fréchet distribution function onto effluent tracer test results from HSFCW-C (Dittrich and Klincsik 2015a). Although the Fréchet distribution has good fittings (Dittrich et al. 2020), this method has the disadvantage that the dispersion coefficient cannot be calculated from the fitted Fréchet distribution curve. Therefore, our aim was to develop another method that would provide a solution.

The most popular method for fitting curves to measured response data is the convection-dispersion transport (CDT) model. The 1D equation of the CDT model is

1 $$ \frac{\partial C}{\partial t}={D}_x\frac{\partial^2C}{\partial {x}^2}-\frac{q}{\varepsilon}\frac{\partial C}{\partial x} $$

where *D*_x_ [m^2^/h] is the longitudinal dispersion coefficient, *q* [1/h] is the specific hydraulic loading rate, and *ε* [-] is the porosity at the time of analysis. The analytical solution of Eq. 1 is with the presumption of a Dirac impulse function at the dose point of the tracer test (Kovács et al. 2002):

2 $$ C\left(x,t\right)=\frac{M}{2\bullet w\bullet m\bullet {\varepsilon}_0\bullet \sqrt{\pi \bullet {D}_x\bullet t}}\bullet {e}^{\frac{{\left(x- vt\right)}^2}{4{D}_xt}} $$

The parameters of Eq. 2 are as follows: *M* [g] is the mass of injected tracer; *w* [m] is the width of seepage zone; *m* [m] is the height of seepage zone; *ε*_0_ [-] is the porosity of filter media; *t* [h] is the elapsed time from impulse moment; *x* [m] is the main direction; and *v*_x_ [m/h] is the velocity in porous regime.

A three-parameter inverse Gaussian distribution function is shown below:

3 $$ g(t)=\frac{1}{2}\sqrt{2}\sqrt{\frac{a}{\pi \bullet {\left(t-c\right)}^3}{e}^{\frac{1}{2}\bullet \frac{a{\left(t-c-b\right)}^Z}{\left(\Big[t-c\right)\bullet b\Big]{}^Z}}\ } $$

where *a* and *b* are the parameters of the distribution function.

The mean and standard deviation of the three-parameter inverse Gaussian distribution function are as follows:

4 $$ \mu =b+c $$

5 $$ \sigma =b\bullet \sqrt{\frac{b}{a}} $$

In this function, parameter *c* represents delay, so it can substitute for the *R* parameter that is mathematically separable. With this solution, a parameter is “exempt” from *R*, so the mean is supplemented:

6 $$ a=\frac{L^2}{2\bullet {D}_x} $$

7 $$ c+b=\frac{L}{v_x} $$

From parameter *c*, the approximate value of *R* can be calculated as follows:

8 $$ R=\frac{c+b}{b} $$

From Eqs. 6, 7, and 8, the *v*_x_, *D*_x_, and *R* parameters can be calculated directly if we can find a good fit for the three-parameter inverse Gaussian distribution curve. This method will be used below for the calculation of transport parameters.

Our model was developed in MAPLE environment; this model fits an inverse Gaussian (IG) distribution function (1st IG curve) onto the main stream and fits the second IG curve onto the side stream while optimizing the main and side stream ratios. For this procedure, we defined parameter *s*; this value shows the ratio of the first IG curve and the D-CDT curve. Our mathematical method that fits a Fréchet distribution function onto the tracer test curve (Dittrich and Klincsik 2015b) was unified in order to obtain a mathematically stable and fast process.

The fitting criteria for the model were as follows:

- At the beginning of the measurement, the tracer concentration is zero all over the CW:

9 $$ \mathrm{if}\kern.5em {\mathrm{t}}_0=0\kern.2em \mathrm{then}\kern.2em {C}_0=0 $$

- The tracer concentration in time converges to zero:

10 $$ \mathrm{if}\kern.5em t\to \infty \mathrm{then}\kern.2em {C}_{\infty}\to 0 $$

- The delay (*c*_1_) of the first IG curve is equal to the best fitted Fréchet distribution function.
- The delay (*c*_2_) of the second IG curve is equal to or higher than the delay of the first IG curve.
- The sum of the first and second IG curves has to give the best fitting function (divided convective-dispersive transport (D-CDT) function) (*R*^2^ → 1).
- The area below the D-CDT function has to be equal to the area of 100% tracer return.
  $$ A\left(\mathrm{G}1\right)+A\left(\mathrm{G}2\right)=M/Q $$

Our model is described in more detail in the article of Dittrich and Klincsik (2015b).

## Results and analysis

**Fig. 2:**
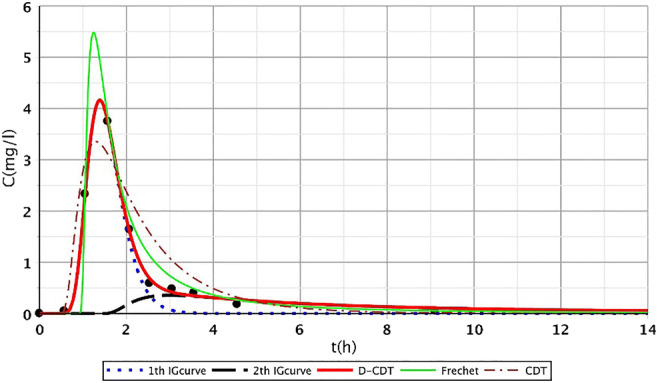
Fit1ting results of the three models (D-CDT, Fréchet distribution, and conventional CDT model) compared with each other on S/1 measurement VII bottom point (black dots are the measurement points)

In this article, the model presented (Dittrich and Klincsik 2015b) was fitted onto inner points (Fig. 2, points I–IX) of all measurements (S/1, S/2, S/3, S/4). Samples were taken at each inner point at top and bottom levels as we assumed that the transport processes at the top and the bottom would differ. Our aim was to prove that the model gives good fittings at the inner points as well and that our hypothesis is also valid for inner points. Our results provide a deeper understanding of transport processes inside the system and give us a more detailed insight into hydrodynamics of CWs.

All fitted curves had better fitting features than the very well-fitting Fréchet distribution. Even the best curve of the Fréchet distribution had less favorable fitting features than those of our model. Table 1 shows *R*^2^ values for the three model types.

**Table 1:** *R*^2^ values of Fréchet distribution, conventional CDT model, and the developed D-CDT model

Ref. number	Fréchet distribution	CDT model	D-CDT model	Ref. number	Fréchet distribution	CDT model	D-CDT model
S/1							
I_t_	1,000	0,999	1,000	I_b_	0,999	0,991	1,000
II_t_	0,995	0,999	1,000	II_b_	0,982	0,992	1,000
III_t_	0,993	0,999	1,000	III_b_	0,988	0,998	1,000
IV_t_	1,000	1,000	1,000	IV_b_	0,993	0,996	1,000
V_t_	1,000	0,836	1,000	V_b_	1,000	0,999	1,000
VI_t_	0,991	0,997	1,000	VI_b_	0,973	0,992	1,000
VII_t_	0,977	0,983	0,988	VII_b_	0,965	0,967	0,998
VIII_t_	0,926	0,935	0,993	VIII_b_	0,944	0,942	0,997
IX_t_	0,999	0,993	0,999	IX_b_	0,998	0,984	0,999
Average	0,987	0,971	0,998	Average	0,983	0,984	0,999
S/2							
I_t_	0,998	0,993	0,996	I_b_	1,000	1,000	1,000
II_t_	0,977	0,973	0,976	II_b_	0,989	0,990	0,996
III_t_	0,997	0,997	1,000	III_b_	0,917	0,161	0,921
IV_t_	0,988	0,956	0,987	IV_b_	0,831	0,864	0,861
V_t_	0,975	0,975	0,991	V_b_	0,970	0,970	0,996
VI_t_	1,000	0,997	0,998	VI_b_	1,000	0,999	0,999
VII_t_	0,976	0,986	0,996	VII_b_	0,994	0,988	0,997
VIII_t_	0,955	0,972	0,993	VIII_b_	0,967	0,968	0,995
IX_t_	0,999	0,987	0,998	IX_b_	0,989	0,982	0,989
Average	0,985	0,979	0,993	Average	0,962	0,880	0,973
S/3							
I_t_	0,980	0,988	0,990	I_b_	0,982	0,981	0,990
II_t_	0,995	0,986	0,996	II_b_	0,807	0,963	0,970
III_t_	0,990	0,986	0,997	III_b_	0,829	0,936	0,944
IV_t_	0,793	0,802	0,975	IV_b_	0,512	0,714	0,833
V_t_	0,773	0,790	0,994	V_b_	0,928	0,973	0,975
VI_t_	0,775	0,795	0,992	VI_b_	0,844	0,913	0,933
VII_t_	0,822	0,925	0,972	VII_b_	0,926	0,940	0,966
VIII_t_	0,986	0,987	0,987	VIII_b_	0,877	0,908	0,977
IX_t_	0,995	0,997	0,999	IX_b_	0,950	0,990	0,991
Average	0,901	0,917	0,989	Average	0,851	0,917	0,953
S/4							
I_t_	0,941	0,810	0,964	I_b_	0,982	0,979	0,990
II_t_	0,980	0,945	0,979	II_b_	0,995	0,968	0,991
III_t_	0,960	0,870	0,990	III_b_	0,994	0,995	0,991
IV_t_	0,953	0,935	0,989	IV_b_	0,983	0,950	0,994
V_t_	0,966	0,977	0,989	V_b_	0,995	0,992	0,999
VI_t_	0,956	0,973	0,981	VI_b_	0,989	0,986	0,999
VII_t_	0,824	0,864	0,973	VII_b_	0,940	0,935	0,989
VIII_t_	0,898	0,941	0,955	VIII_b_	0,897	0,823	0,975
IX_t_	0,819	0,943	0,993	IX_b_	0,966	0,968	0,990
Average	0,922	0,918	0,979	Average	0,971	0,955	0,991

We chose fitting figures that demonstrate considerable differences among the three functions.

Figures 2, 3, 4, 5, 6, 7, 8, 9, and 10 show the fitting results of the D-CDT model compared with the Fréchet distribution and a conventional CDT model. All images of fitting results are shown in Appendix 3 (Figs. 12, 13, 14, 15, 16 and 17).

**Fig. 3:**
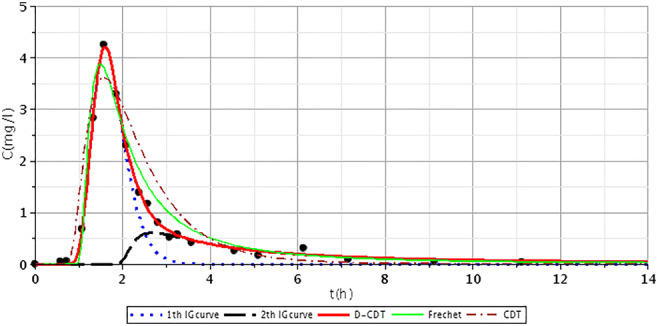
Fitting results of the three models (D-CDT, Fréchet distribution, and conventional CDT model) compared with each other on S/1 measurement VIII bottom point (black dots are the measurement points)

**Fig. 4:**
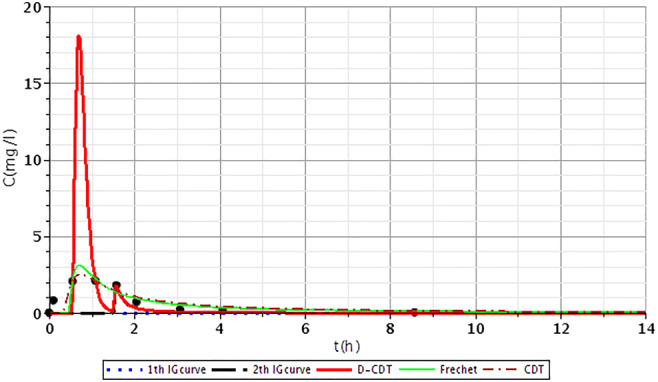
Fitting results of the three models (D-CDT, Fréchet distribution, and conventional CDT model) compared with each other on S/2 measurement IV bottom point (black dots are the measurement points)

**Fig. 5:**
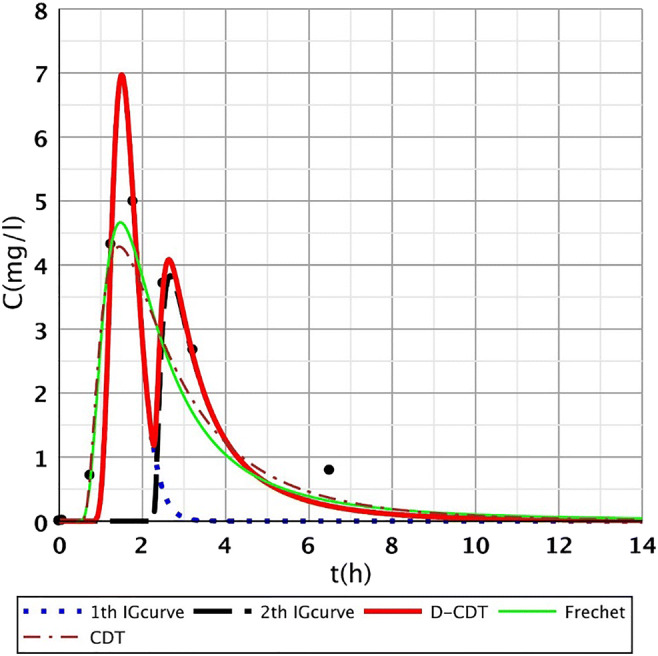
Fitting results of the three models (D-CDT, Fréchet distribution, and conventional CDT model) compared with each other on S/3 measurement IV top point (black dots are the measurement points)

**Fig. 6:**
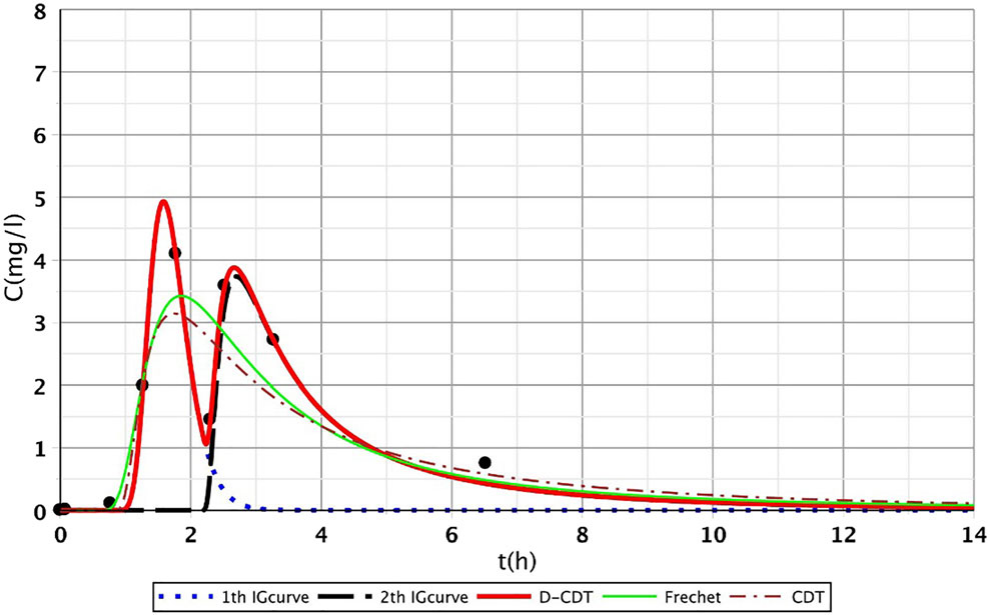
Fitting results of the three models (D-CDT, Fréchet distribution, and conventional CDT model) compared with each other on S/3 measurement V top point (black dots are the measurement points)

**Fig. 7:**
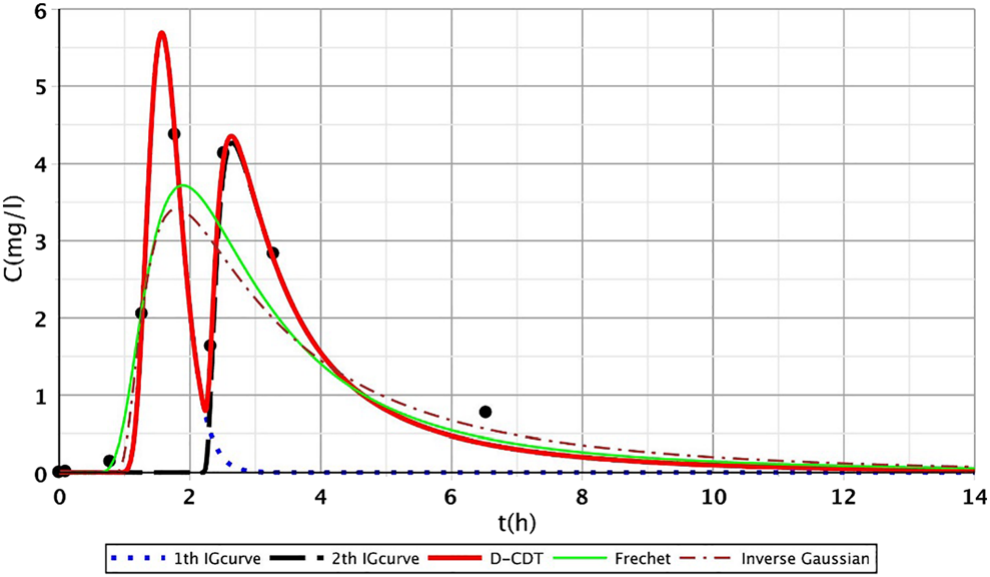
Fitting results of the three models (D-CDT, Fréchet distribution, and conventional CDT model) compared with each other on S/3 measurement VI top point (black dots are the measurement points)

**Fig. 8:**
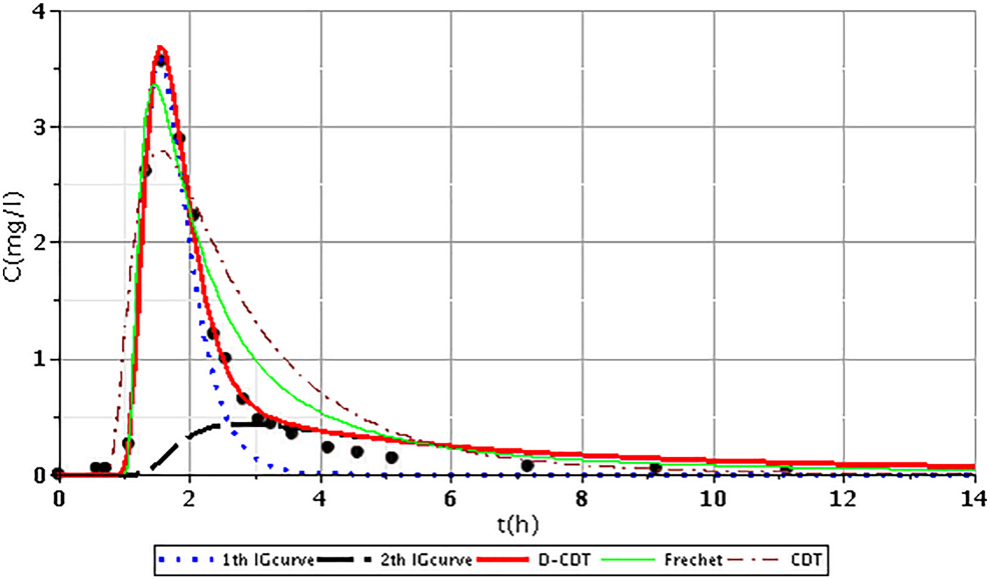
Fitting results of the three models (D-CDT, Fréchet distribution, and conventional CDT model) compared with each other on S/1 measurement VIII top point (black dots are the measurement points)

**Fig. 9:**
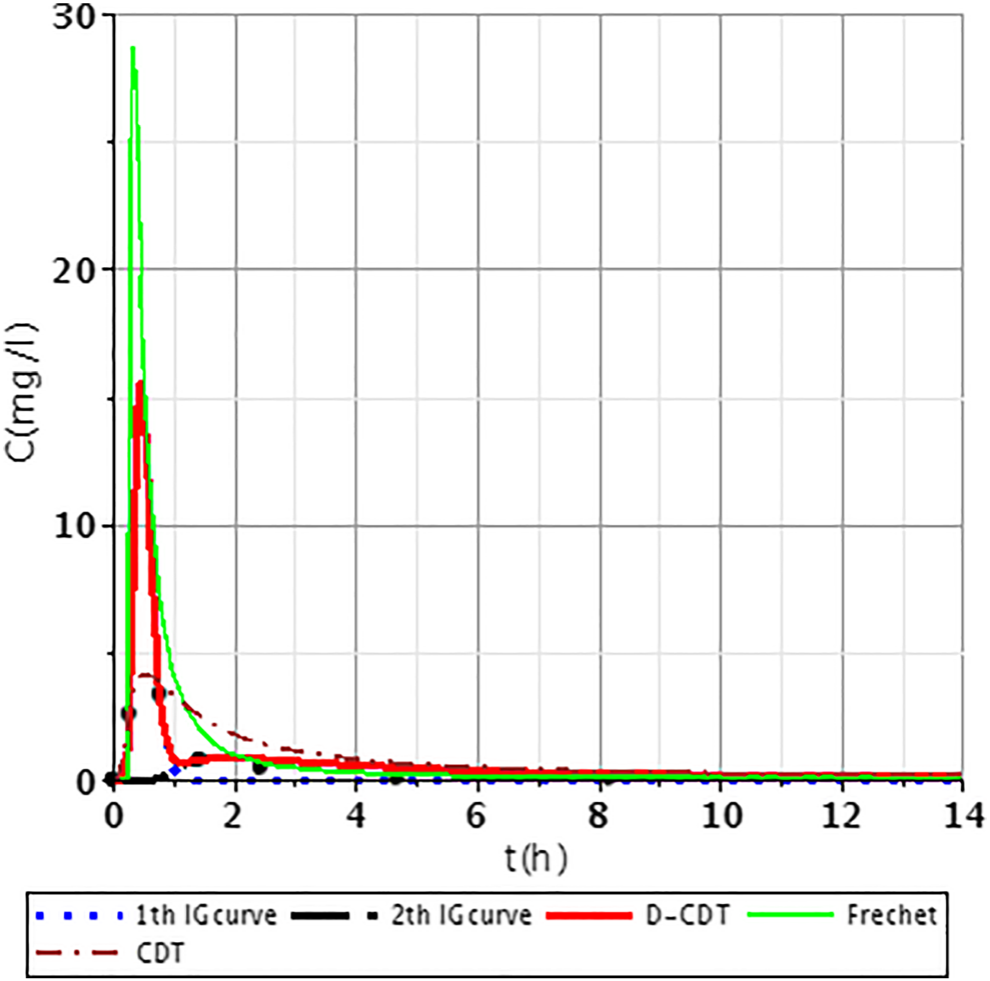
Fitting results of the three models (D-CDT, Fréchet distribution, and conventional CDT model) compared with each other on S/4 measurement I top point (black dots are the measurement points)

**Fig. 10:**
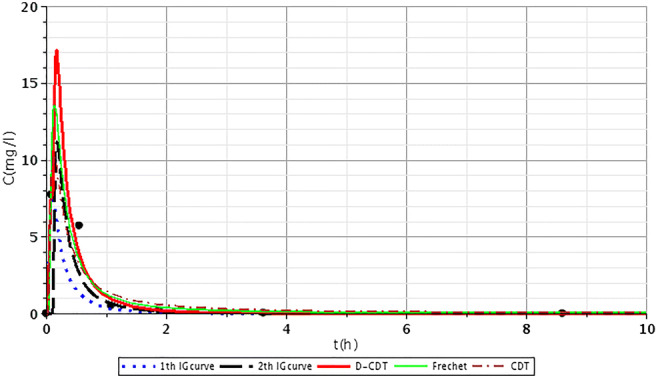
Fitting results of the three models (D-CDT, Fréchet distribution, and conventional CDT model) compared with each other on S/2 measurement III bottom point (black dots are the measurement points)

The red curves on Figs. 2, 3, 4, 5, 6, 7, 8, 9, and 10 show the very nice fitting properties of the D-CDT model. The effect of the main stream from the blue curves and the effect of the side streams from black curves can be seen.

Using Eqs. (6, 7 and 8), the *v*_*x*_, *D* and *R* of the 1st and 2nd IG curves were calculated. Subsequently, the average *R* and *v*_x_ values were calculated and weighted by the areas of IG curves. Calculated results are summarized in Tables 3 and 4. The average velocity means the theoretical value (homogeneous flow regime). The fitting parameters of the measurement S/1–S/4 are summarized in Appendix 2 (Tables 12, 13, 14, 15, 16, 17, 18, and 19).

## Discussion

### Fitting results

Table 1 demonstrates that the D-CDT model gives better fitting than the Fréchet distribution or the CDT model at all points. All average *R*^2^ values were above 0.96. The S/4 top measurement results showed the biggest differences in *R*^2^ values of the D-CDT model (0.97) and the CDT model (0.88).

The S/1 measurement (Table 1) gave the best fitting results of all 4 measurements. This is mainly due to the fact that there was no hydraulic distortion in the CW, as neither the roots nor the formation of the biofilm had any impact upon flow processes. The results show that the D-CDT model has better fittings than the Fréchet distribution and even better than the conventional CDT model fittings.

Upon the S/2 measurement (Table 1), the CW was 1-month old as the results also demonstrate. Even so, almost all *R*^2^ results yielded by the D-CDT model were higher than 0.95. There was an extreme hydraulic short circuit at the fourth bottom measuring point resulting in a very bad fitting; nevertheless, the D-CDT model provided a better fitting than the CDT model and the Fréchet distribution. As a result of this bad fitting, our model needs to be further developed and extended with a short circuiting part.

Upon the S/3 measurement (Table 1), similar fitting results were obtained (D-CDT model) as upon the second, but the results of the CDT model and Fréchet distribution were considerably worse. For the third, fourth, and sixth bottom measuring points, there were extreme hydraulic short circuits that resulted in very bad fittings. Nonetheless, the D-CDT model proved to provide much better fitting than the CDT model or the Fréchet distribution.

Upon the fourth measurement (Table 1), the results of the three functions showed differences. The D-CDT model gave better fittings than the other two methods. This was especially true at the first, third, and seventh top points, where the CDT model showed very bad fittings and the D-CDT model had especially good fittings.

### Macro- and micro-porous ratio

When comparing the *s* values (Table 2), the top value was similar at S/1 and S/2 measurements, while the value of *s* was reduced at S/3 and S/4 measurements. Overall, it can be said that the rate of micro-porous system volume to total porous volume grew over time from 38 to 57%.

**Table 2:** Average s values of the measurements

Measurement	Average *s* value
S/1. Top	0,62
S/1. Bottom	0,67
S/2. Top	0,62
S/2. Bottom	0,61
S/3. Top	0,54
S/3. Bottom	0,58
S/4. Top	0,43
S/4. Bottom	0,51

The *s* values of the bottom measurements presented a gradual decrease until the S/4 measurement. In general, it can also be observed that with regard to the bottom measuring points, the rate of micro-porous system volume to the total porous volume grew over time from 32,9 to 49%.

### The role of the main stream and side stream

At the S/1 top and bottom points (Table 3), the side stream had a higher dispersion coefficient than the main stream. It showed significant back mixing in this HSFCW. At points IV, V, VI, VII, and IX, the weighted velocity value was higher than the bottom values which is possible because the CW was 1 day old and the roots and biofilm activity did not affect transport processes.

**Table 3:** Calculated transport parameters at S/1 and S/2 top and bottom points based on the fitted D-CDT model

Ref. number	Average *v*_x_ (m/h)	1st inverse Gaussian curve				2nd inverse Gaussian curve				weighted *v*_x_ (m/h)	Weighted *R* (-)
		*v*_x1_ (m/h)	*D*_1_ (m^2^/d)	*D*_i_ (-)	*R*_1_ (-)	*v*_x2_ (m/h)	*D*_2_ (m^2^/d)	*D*_i_ (-)	*R*_2_ (-)		
S/1											
I_t_	3,49	1,73	1,79	0,05	1,25	0,44	3,31	0,08	1,37	0,95	1,32
II_t_	3,49	1,16	2,52	0,06	1,04	0,38	2,73	0,07	2,71	0,89	1,63
III_t_	3,49	1,58	1,38	0,04	1,48	0,46	12,85	0,33	2,83	1,20	1,94
IV_t_	3,49	1,48	26,37	0,14	1,33	0,88	39,75	0,21	1,60	1,24	1,44
V_t_	3,49	1,00	3,07	0,02	1,01	0,16	4,63	0,03	1,07	0,83	1,02
VI_t_	3,49	1,32	1,92	0,01	1,01	0,62	3,80	0,02	1,73	1,15	1,19
VII_t_	3,49	2,68	11,50	0,04	1,14	0,44	40,42	0,12	1,06	1,90	1,12
VIII_t_	3,49	2,17	26,75	0,08	1,66	0,31	30,72	0,09	1,08	1,20	1,36
IX_t_	3,49	2,94	4,95	0,02	1,17	1,20	327,13	1,01	2,14	2,24	1,56
I_b_	3,49	1,89	2,95	0,07	1,19	0,28	3,47	0,09	1,74	1,17	1,44
II_b_	3,49	1,16	2,52	0,06	1,04	0,39	7,20	0,18	2,14	0,89	1,43
III_b_	3,49	2,02	0,78	0,02	1,16	0,47	1,26	0,03	1,70	1,44	1,36
IV_b_	3,49	1,23	9,83	0,05	1,24	0,51	25,98	0,14	2,31	1,08	1,45
V_b_	3,49	1,03	2,57	0,01	1,00	0,26	28,15	0,15	1,47	0,78	1,16
VI_b_	3,49	1,28	1,97	0,01	1,00	0,47	27,71	0,15	2,75	1,08	1,44
VII_b_	3,49	2,48	10,46	0,03	1,08	0,31	33,55	0,10	1,11	1,61	1,09
VIII_b_	3,49	2,21	8,50	0,03	1,18	0,32	63,68	0,20	1,17	1,40	1,18
IX_b_	3,49	2,39	8,10	0,02	1,14	1,24	33,12	0,10	1,74	2,16	1,26
S/2											
I_t_	4,33	1,28	1,56	0,03	1,26	0,08	28,12	0,58	1,04	0,76	1,16
II_t_	4,33	0,66	5,40	0,11	1,06	0,07	5,69	0,12	1,03	0,45	1,05
III_t_	4,33	1,95	1,61	0,03	1,10	0,57	8,93	0,18	3,55	1,63	1,66
IV_t_	4,33	3,02	35,56	0,15	1,07	0,17	41,24	0,18	1,15	2,16	1,10
V_t_	4,33	2,96	16,99	0,07	1,13	0,22	21,53	0,09	1,03	1,43	1,07
VI_t_	4,33	3,17	15,62	0,07	1,21	0,04	17,50	0,08	1,01	2,14	1,14
VII_t_	4,33	4,95	28,20	0,07	1,03	2,56	31,37	0,08	1,64	4,23	1,21
VIII_t_	4,33	2,93	22,35	0,06	1,21	0,38	32,84	0,08	1,05	1,63	1,13
IX_t_	4,33	3,77	32,34	0,08	1,56	2,08	51,66	0,13	1,60	3,09	1,57
I_b_	4,33	0,63	1,22	0,03	1,16	0,19	2,99	0,06	1,47	0,54	1,22
II_b_	4,33	0,68	1,72	0,04	1,01	0,07	2,06	0,04	1,08	0,50	1,03
III_b_	4,33	0,93	6,76	0,14	1,01	0,91	9,35	0,19	1,22	0,92	1,09
IV_b_	4,33	2,26	23,69	0,10	1,00	0,00	25,36	0,11	1,00	0,99	1,00
V_b_	4,33	4,03	19,14	0,08	1,20	0,07	86,82	0,38	1,02	1,42	1,08
VI_b_	4,33	3,20	19,22	0,08	1,25	0,43	35,56	0,15	1,10	2,18	1,19
VII_b_	4,33	3,49	37,76	0,09	1,23	0,14	67,01	0,17	1,04	2,18	1,15
VIII_b_	4,33	2,83	16,08	0,04	1,05	0,48	41,46	0,10	1,18	1,98	1,10
IX_b_	4,33	3,65	9,63	0,02	1,01	2,02	36,17	0,09	1,67	3,11	1,23

In Table 3, at the S/2 top and bottom points, the side stream showed a higher dispersion coefficient than the main stream, same as at S/1. At points I, III, IV, V, and VII, the weighted velocity value was higher than the bottom values. At points II, VI, VIII, and IX, these values were the opposite. It means that the roots and the biofilm had been growing and were consequently affecting transport processes. This was possible as the CW was 1 month old. In the cross-section, the middle points (II, V, VIII) showed the lowest velocity values, which led us to the conclusion that it was the root system and biofilm that had the greatest effect on the flow in the middle section from which it follows that the main flow path was on the two sides.

Upon the S/3 measurement showed in Table 4, the CW was 5 months old. The results present that at both the top and bottom points, the side stream had a higher dispersion coefficient than the main stream. At bottom points I, III, VI, VII, and IX, the weighted velocity values were higher than the top values. In the cross-section, the points II, VI, and IX had the lowest velocity values; this means that the root system and biofilm had the most significant impact upon the flow at these points. Consequently, the main flow path was at points III, VI, and VII.

**Table 4:** Calculated transport parameters at S/3 and S/4 top and bottom points based on the fitted D-CDT model

Ref. number	Average *v*_x_ (m/h)	1st inverse Gaussian curve				2nd inverse Gaussian curve				Weighted *v*_x_ (m/h)	Weighted *R* (-)
		*v*_x1_ (m/h)	*D*_1_ (m^2^/d)	*D*_i_ (-)	*R*_1_ (-)	*v*_x2_ (m/h)	*D*_2_ (m^2^/d)	*D*_i_ (-)	*R*_2_ (-)		
S/3											
I_t_	3,18	0,37	0,91	0,03	1,16	0,11	1,43	0,04	1,80	0,31	1,32
II_t_	3,18	0,43	0,28	0,01	1,21	0,12	0,61	0,02	1,26	0,25	1,24
III_t_	3,18	0,43	0,27	0,01	1,16	0,11	0,87	0,02	1,66	0,28	1,40
IV_t_	3,18	1,34	4,45	0,03	1,42	0,56	36,54	0,22	2,23	0,93	1,85
V_t_	3,18	1,29	4,11	0,02	1,51	0,79	32,33	0,19	1,05	0,95	1,19
VI_t_	3,18	1,32	3,87	0,02	1,58	0,50	36,70	0,22	1,95	0,76	1,83
VII_t_	3,18	1,24	6,07	0,02	1,00	0,74	19,29	0,07	3,07	1,09	1,62
VIII_t_	3,18	1,00	8,05	0,03	1,18	0,49	12,67	0,04	1,55	0,97	1,21
IX_t_	3,18	0,86	7,17	0,02	1,09	0,19	59,47	0,20	1,42	0,72	1,15
I_b_	3,18	0,49	0,37	0,01	1,07	0,11	6,38	0,18	1,46	0,39	1,17
II_b_	3,18	0,30	0,20	0,01	1,18	0,10	1,31	0,04	2,48	0,19	1,88
III_b_	3,18	0,53	0,79	0,02	1,24	0,26	7,54	0,21	9,67	0,51	1,91
IV_b_	3,18	2,75	13,25	0,08	1,17	0,49	18,52	0,11	1,52	0,72	1,48
V_b_	3,18	1,07	2,99	0,02	1,11	0,53	12,24	0,07	2,11	0,81	1,59
VI_b_	3,18	2,66	2,22	0,01	2,94	0,60	10,04	0,06	2,57	1,67	2,76
VII_b_	3,18	1,30	7,17	0,02	1,23	0,71	34,65	0,12	2,54	1,01	1,88
VIII_b_	3,18	1,28	6,69	0,02	1,35	0,62	21,27	0,07	1,76	0,80	1,65
IX_b_	3,18	0,82	5,60	0,02	1,02	0,59	8,13	0,03	1,62	0,79	1,11
S/4											
I_t_	2,70	0,89	0,45	0,01	1,01	0,01	0,57	0,02	1,01	0,36	1,01
II_t_	2,70	0,99	5,20	0,17	1,00	1,02	**0**	**0**	1,60	1,01	1,48
III_t_	2,70	0,95	0,76	0,02	1,47	0,00	3,70	0,12	1,00	0,72	1,36
IV_t_	2,70	0,95	0,76	0,02	1,47	0,00	3,70	0,12	1,00	0,72	1,36
V_t_	2,70	2,03	8,49	0,06	2,00	0,03	56,54	0,39	1,02	1,47	1,73
VI_t_	2,70	1,23	8,47	0,06	1,20	0,13	8,60	0,06	1,04	0,66	1,12
VII_t_	2,70	1,60	7,40	0,05	1,08	0,11	27,16	0,19	1,07	0,74	1,08
VIII_t_	2,70	1,76	14,67	0,06	1,18	0,01	17,80	0,07	1,00	0,59	1,06
IX_t_	2,70	1,57	14,78	0,06	1,31	0,07	19,36	0,08	1,03	0,52	1,12
I_b_	2,70	0,37	0,59	0,02	1,02	0,04	2,31	0,08	1,26	0,29	1,08
II_b_	2,70	0,77	1,08	0,04	1,33	0,06	4,96	0,16	1,00	0,15	1,04
III_b_	2,70	0,85	1,39	0,05	1,00	0,02	1,90	0,06	1,02	0,48	1,01
IV_b_	2,70	2,59	9,43	0,07	1,61	0,46	35,09	0,25	1,40	1,95	1,55
V_b_	2,70	1,32	26,87	0,19	1,38	0,18	31,05	0,22	1,09	0,92	1,28
VI_b_	2,70	1,31	9,85	0,07	1,11	0,16	10,10	0,07	1,17	0,90	1,13
VII_b_	2,70	1,68	12,51	0,05	1,08	0,03	21,44	0,09	1,02	0,77	1,05
VIII_b_	2,70	1,56	10,19	0,04	1,44	0,34	21,74	0,09	1,22	0,76	1,30
IX_b_	2,70	1,70	15,19	0,06	1,37	0,25	19,10	0,08	1,09	0,84	1,21

The results gained upon the last measurement (the CW was 9 months old) are shown in Table 4. At both the top and bottom points, the side stream had a higher dispersion coefficient than the main stream, similar to S/1. At points I, II, and III, the top weighted velocity values were higher than the bottom values; therefore, the main flow path was in the first section on the top; at points IV–IX, this was the opposite. This phenomenon is due to the fact that the root system on the top was located much higher and the biofilm activity was much greater than at the bottom. Similar to the results observed by Liu et al. (2018), this resulted in the so-called bottom flow effect, meaning that the main flow path was at the bottom and at the top there was some back mixing, short circuits, and dead zones. Upon all measurements, the preferred transport path is at the influent and effluent regions of the CWs.

**Fig. 11:**
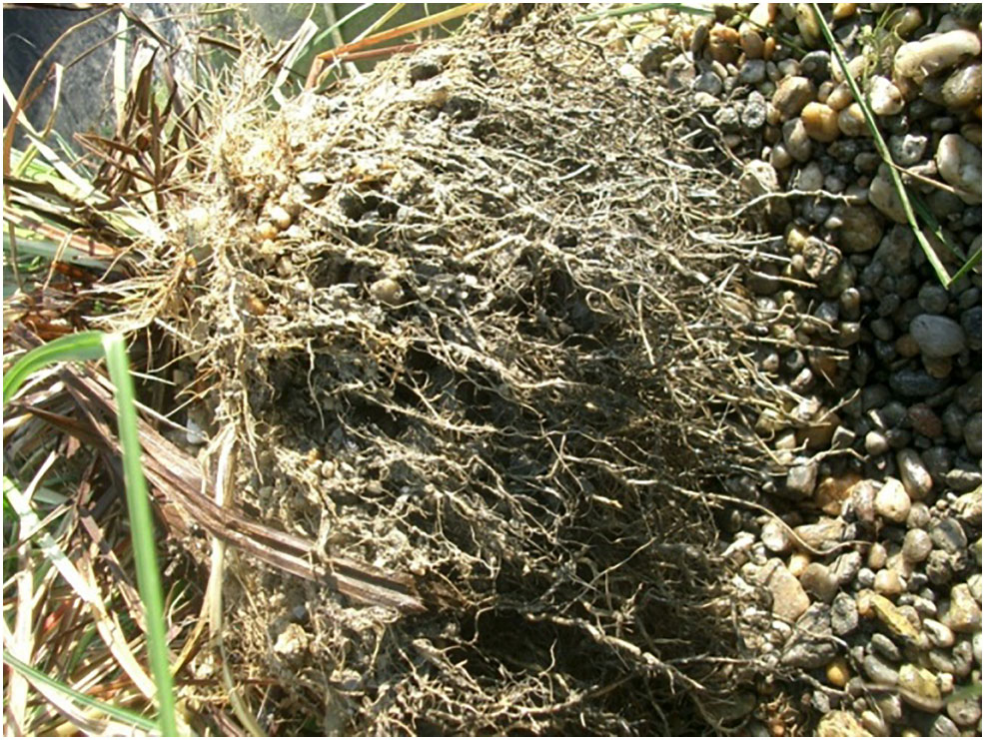
The rhizome systems of sedge form island-like zones in the medium

The reasons for significant back mixing in this CW were as follows:

- The distribution pipe was inadequately positioned. The pipe was approximately 0.8 m from the planned place in the direction of the flow, so wastewater could flow back under the distribution pipe.
- The seated *Carex acutiformis* has a globular root system (see Fig. 11); back mixing zones can develop behind these well-defined root zones. This phenomenon is caused by the smaller hydraulic conductivity of fields with high root density, as in such cases the wastewater needs to change flow direction in the filter media.

**Fig. 12:**
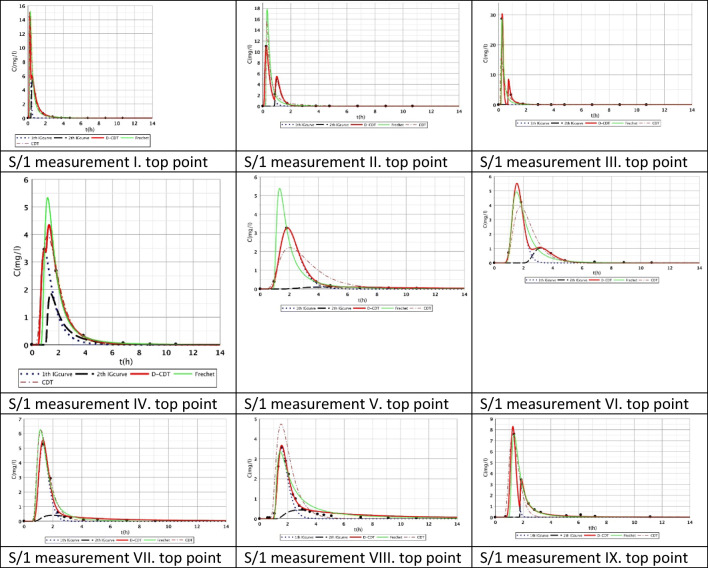
Fitting results of D-CDT model compared with Fréchet distribution and conventional CDT model on S/1 measurement top points

Our results showed that upon all measurements, the theoretical velocity was higher than the weighted average. This means that the actual HRT was higher than the theoretical HRT (Dittrich and Klincsik 2015b), a result that contradicts results in international literature (Schierup et al. 1990; Breen and Chick’s 1995; Tanner and Sukias 1995; King et al. 1997). The values of R are between 1.00–2.67, which show the extent of dead zones in the systems.

### Comparison of main stream D-CDT model with conventional CDT model

Table 5 shows the comparison of the main stream D-CDT and the conventional CDT model based on S/1 top and bottom point measurements. At all points, the D-CDT model provided lower porous velocity and higher dispersion coefficient values than the CDT model, with significant differences in some cases. The velocities were 8–71% lower, and dispersion coefficients were 0–352% higher.

**Table 5:** Comparison of D-CDT main stream and conventional CDT model S/1 and S/2 top and bottom points

Ref. number	*v* (m/h)	*v*_x1_ (m/h)	Difference (%)	*D* (m^2^/day)	*D*_1_ (m^2^/day)	Difference (%)
S/1						
I_t_	0,74	1,73	57,45	5,83	1,79	− 225,32
II_t_	0,82	1,16	29	6,45	2,52	− 155,79
III_t_	1,49	1,58	5,91	3,45	1,38	− 150
IV_t_	1,22	1,48	17,43	26,37	26,37	0
V_t_	0,91	1	8,59	3,27	3,07	− 6,63
VI_t_	1,02	1,32	22,73	6,51	1,92	− 239,61
VII_t_	2,47	2,68	7,91	43,8	11,5	− 280,88
VIII_t_	1,33	2,17	38,7	49,31	26,75	− 84,35
IX_t_	2,49	2,94	15,05	18,91	4,95	− 282,11
I_b_	0,53	1,89	71,99	12,67	2,95	− 329,90
II_b_	0,68	1,16	41,41	3,99	2,52	− 58,3
III_b_	1,46	2,02	27,47	3,24	0,78	− 313,07
IV_b_	0,97	1,23	20,57	10,13	9,83	− 3,11
V_b_	0,92	1,03	10,96	7,51	2,57	− 191,5
VI_b_	1,01	1,28	21,52	7,52	1,97	− 281,69
VII_b_	1,71	2,48	31,13	47,27	10,46	− 352,06
VIII_b_	1,67	2,21	24,7	36,13	8,5	− 325,16
IX_b_	2,2	2,39	8,16	21,4	8,1	− 164,12
S/2						
I_t_	1,22	1,28	4,36	3,08	1,56	− 96,75
II_t_	0,26	0,66	60,9	6,02	5,4	− 11,46
III_t_	1,41	1,95	27,63	5,96	1,61	− 270,67
IV_t_	0,91	3,02	69,8	57,01	35,56	− 60,34
V_t_	0,90	3,01	69,97	106,58	25,09	− 324,78
VI_t_	3,23	3,17	− 2,02	51,29	15,62	− 228,43
VII_t_	3,42	4,95	30,83	62,67	28,2	− 122,22
VIII_t_	1,42	2,93	51,64	93,71	22,35	− 319,25
IX_t_	3,25	3,77	13,85	52,16	32,34	− 61,29
I_b_	0,53	0,63	15,53	1,58	1,22	− 29,03
II_b_	0,38	0,68	44,07	3,13	1,72	− 82,54
III_b_	0,24	0,93	74,41	7,97	6,76	− 17,96
IV_b_	0,39	2,26	82,52	38,24	23,69	− 61,44
V_b_	0,87	4,03	78,56	94,66	19,14	− 394,66
VI_b_	3,19	3,2	0,4	38,03	19,22	− 97,88
VII_b_	2,01	3,49	42,24	81,08	37,76	− 114,73
VIII_b_	1,89	2,83	33,14	74,42	16,08	− 362,96
IX_b_	2,93	3,65	19,76	35,99	9,63	− 273,5

Upon the S/2 measurement, in most cases (except the VI top point, with a difference of only 2%), the results proved that the D-CDT model provided lower porous velocity and higher dispersion coefficient values than the CDT model as shown in Table 5.The velocities are lower by 0–82%, and dispersion coefficients are higher by 11–394%.

Table 6 shows the comparison of main stream D-CDT and conventional CDT models based on S/3 top and bottom point measurements. The D-CDT model yielded lower porous velocity and higher dispersion coefficient values than the CDT model in most cases (except IV bottom point which showed the worst fitting results); consequently, our model needs to be improved and developed further. The velocities were reduced by 7–81%, a range similar to those at S/1 and S/2, and dispersion coefficients were higher by 2–343%.

**Table 6:** Comparison of D-CDT main stream and conventional CDT model S/3 and S/4 top and bottom points

Ref. number	*v* (m/h)	*v*_x1_ (m/h)	Difference (%)	*D* (m^2^/day)	*D*_1_ (m^2^/day)	Difference (%)
S/3						
I_t_	0,21	0,37	43,1	2,04	0,91	− 125,12
II_t_	0,19	0,43	56,11	1,22	0,28	− 329,51
III_t_	0,22	0,43	48,86	1,1	0,27	− 312,04
IV_t_	0,76	1,34	43,68	15,28	4,45	− 243,29
V_t_	0,51	1,29	60,49	16,64	4,11	− 304,84
VI_t_	0,58	1,32	56,45	15,62	3,87	− 304,25
VII_t_	0,97	1,24	21,59	7,27	6,07	− 19,87
VIII_t_	0,79	1	21,1	18,65	8,05	− 131,57
IX_t_	0,71	0,86	17,03	14,52	7,17	− 102,47
I_b_	0,32	0,49	35,57	1,18	0,37	− 217,72
II_b_	0,16	0,3	46,47	0,29	0,2	− 49,08
III_b_	0,46	0,53	13,73	0,82	0,79	− 3,42
IV_b_	0,52	2,75	81,14	3,06	13,25	76,89
V_b_	0,72	1,07	32,39	7,55	2,99	− 152,56
VI_b_	0,84	2,66	68,59	9,82	2,22	− 343,15
VII_b_	0,91	1,3	29,84	13,11	7,17	− 82,89
VIII_b_	0,7	1,28	45,63	9,37	6,69	− 40,07
IX_b_	0,76	0,82	7,76	5,75	5,6	− 2,53
S/4						
I_t_	0,02	0,84	97,18	1,59	0,45	− 250,73
II_t_	0,08	0,99	92,33	8,17	5,20	− 57,20
III_t_	0	0,95	100	0,79	0,76	− 3,84
IV_t_	0,1	2,03	95,1	16,6	8,49	− 95,55
V_t_	0,47	1,23	61,51	30,51	8,47	− 260,19
VI_t_	0,47	1,6	70,51	26	7,4	− 251,16
VII_t_	0,46	1,76	73,95	50,35	14,67	− 243,26
VIII_t_	0,4	1,57	74,44	43,57	14,78	− 194,83
IX_t_	0,48	1,67	71,29	60,25	13,24	− 355,04
I_b_	0,23	0,37	39,23	1,34	0,59	− 126,14
II_b_	0,51	0,85	39,50	5,33	1,09	− 390,66
III_b_	0,75	0,85	11,84	4,82	1,39	− 247,02
IV_b_	0,25	2,59	90,51	20,58	9,43	− 118,25
V_b_	0,86	1,32	34,97	29,86	26,87	− 11,11
VI_b_	0,73	1,31	43,94	36,92	9,85	− 274,82
VII_b_	0,51	1,68	69,58	51,42	12,51	− 311,18
VIII_b_	0,55	1,56	64,53	46,14	10,19	− 352,71
IX_b_	0,61	1,7	63,83	73,11	15,19	− 381,33

The results of the final measurement are shown in Table 6. At all top and bottom points, the D-CDT model gave lower porous velocity and higher dispersion coefficient values than the CDT model. The velocities were lower by 11–99%, and dispersion coefficients were higher by 11–390%.

### Transport processes controlled by convective-dispersive transport

The diffusion from the perspective of transport processes in the micro-porous system of HSFCW-Cs is not significant. In these systems, there are main stream and side stream regimes with side streams having lower porous velocity and dispersion in the micro-porous system than in the main stream. Nonetheless, it is important to emphasize that these parameters are much higher than diffusion values. In our model, the first inverse Gaussian curve (Figs. 2, 3, 4, 5, 6, 7, 8, 9, and 10; blue dotted line) clearly demonstrates the convective-dispersive transport caused by the main stream. The second inverse Gaussian curve (Figs. 2, 3, 4, 5, 6, 7, 8, 9, and 10; black line) illustrates that the convective-dispersive transport of water slowed down in micro-porous systems. The second curve shows the impact of back mixing and dead zones in the side stream combined. These phenomena are significant from the viewpoint of transport processes in such systems. According to our observations, the micro-porous volume grew over time (Table 2). Biofilm activity and root growth have a significant effect upon transport processes, especially with the aging of constructed wetlands. These factors determine flow directions, dead zones, and short circuits in these CWs (e.g., see Table 3, points II, V, and VII). Our model presents these phenomena much more accurately than currently used models (Tables 5 and 6). Our model needs further improvement, especially at points with extreme hydraulic short circuits as our model yielded bad fittings. Nonetheless, our fittings were considerably better than those achieved by the CDT model for example (Table 1; S/3 III, IV, and VI bottom points). The separation of back mixing, dead zones, and side streams are currently being investigated with our model.

## Conclusions

A divided convective-dispersive transport (D-CDT) model was designed and constructed with the aim to accurately simulate conservative transport processes in planted, horizontal, subsurface flow constructed wetlands filled with coarse gravel (HSFCW-C). This model makes fitted response curves from the sum of two independent CDT curves, while model optimizing the rate between the main stream and the side streams. In our model, the first inverse Gaussian curve clearly demonstrates the convective-dispersive transport caused by the main stream; the second inverse Gaussian curve illustrates that the convective-dispersive transport of water slowed down in a micro-porous system. The second curve integrates the back mixing and effects of dead zones as well. The diffusion is not significant with regard to transport processes in the micro-porous system of HSFCW-Cs. The analytical solutions for these two CDT curves are inverse Gaussian distribution functions. Fréchet distribution was used for the rapid optimization of the mathematical procedure (Dittrich and Klincsik 2015b).

The results show that the model is not only adequate and relevant at the effluent points (Dittrich and Klincsik 2015b), but good fittings were achieved at the inner points as well. The D-CDT model proved to give better fittings than the conventional CDT model (Table 1). There are some fittings that yielded considerable differences between the two models. The results indicated that the ratio of micro-porous system volume to the total porous volume grew over time, at the top from 38 to 57% and at the bottom from 32.9 to 49% (Table 2).

The results also showed that in the 9-month-old CW, the main flow path was at the bottom layers; at the top layer, there were some short circuits and dead zones. Calculated velocity and dispersion coefficients upon using the D-CDT model gave differences of 7–99% (of velocity) and 2–394% (of dispersion coefficient) when compared with the conventional CDT model; results approximated real hydraulic behavior more closely (Tables 5–6).

Overall, it can be said that the designed D-CDT model can be applied to inner points and can thus help discover and understand the hydraulic behavior and transport processes in planted horizontal subsurface flow constructed wetlands.

One of our goals with this fitting procedure in Maple environment was to provide a novel, adaptable method of analysis for other types of hydraulic regimes and, thereby, to aid scientists with their research analyses of transport test results. In our opinion, the outlined statistical method can provide an adequate tool for a deeper understanding of several hydrodynamic problems for the solution of which traditional methods have not been successful, mainly hydraulic leakage problems in other media. Our further research direction is to develop a software that could be available for wider application. One of the main directions of our further research is to find other areas where similar research could be performed with success.

## Figures and Tables

**Fig. 13:**
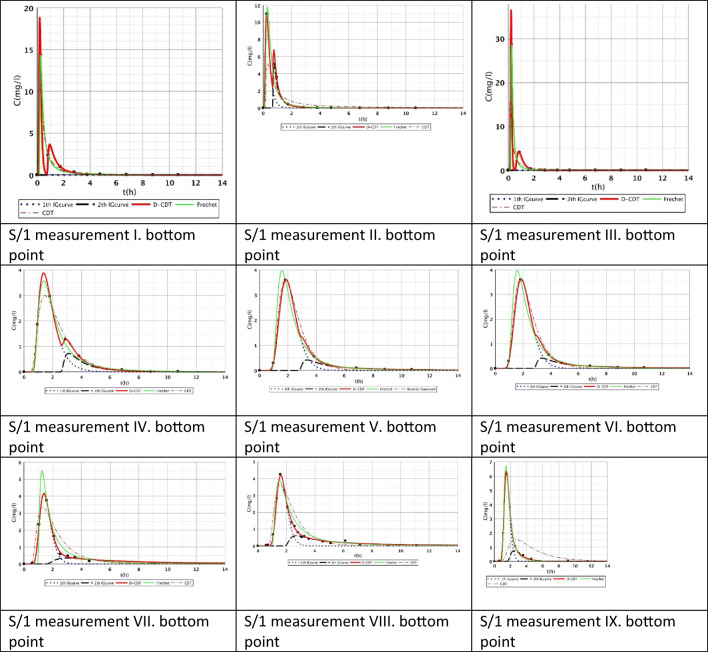
Fitting results of D-CDT model compared with Fréchet distribution and conventional CDT model on S/1 measurement bottom points

**Fig. 14:**
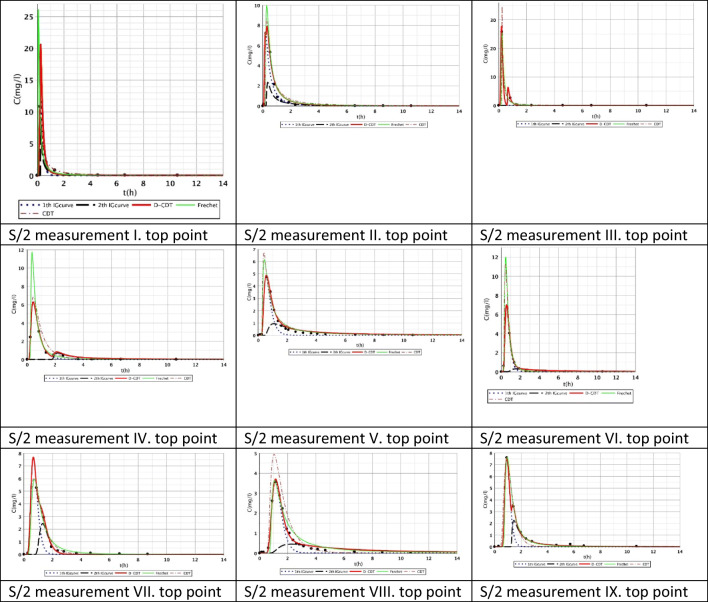
Fitting results of D-CDT model compared with Fréchet distribution and conventional CDT model on S/2 measurement top points

**Fig. 15:**
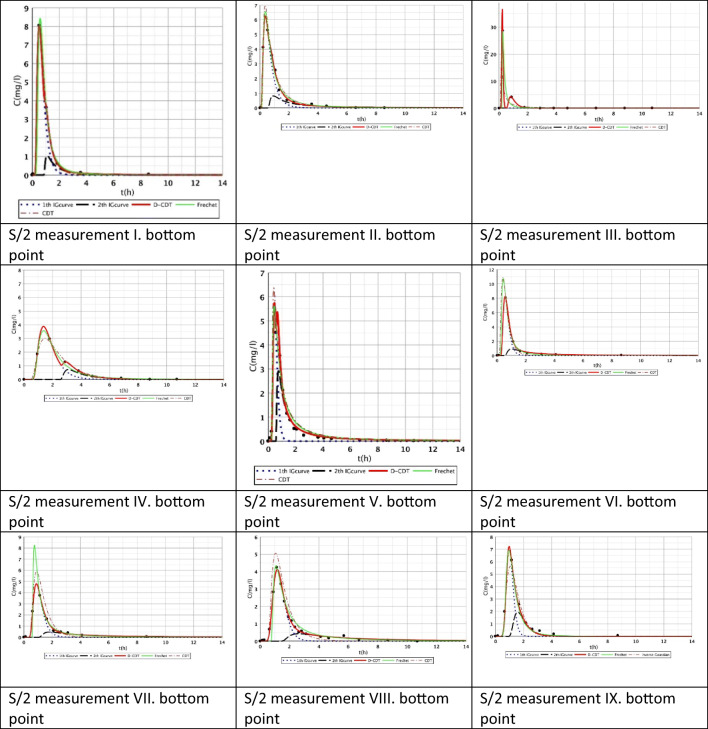
Fitting results of D-CDT model compared with Fréchet distribution and conventional CDT model on S/2 measurement bottom points

**Fig. 16:**
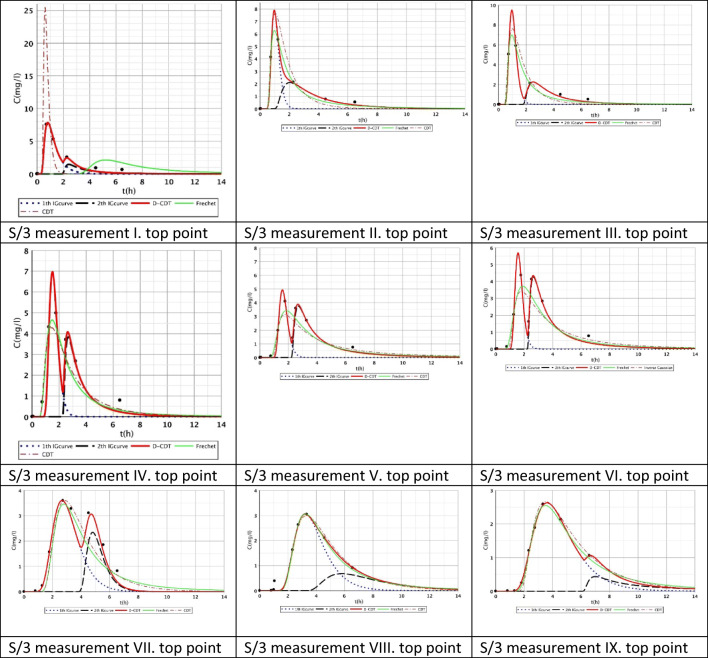
Fitting results of D-CDT model compared with Fréchet distribution and conventional CDT model on S/3 measurement top points

**Fig. 17:**
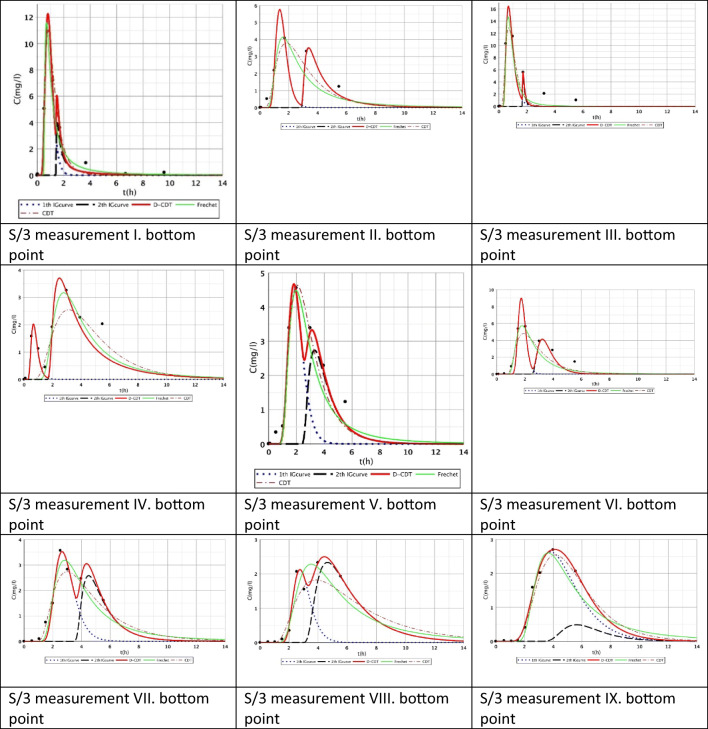
Fitting results of D-CDT model compared with Fréchet distribution and conventional CDT model on S/3 measurement bottom points

**Fig. 18:**
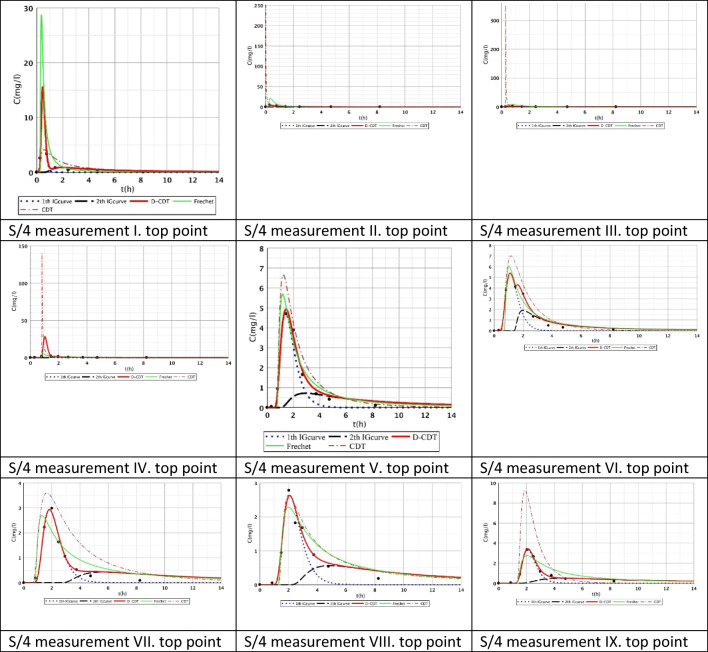
Fitting results of D-CDT model compared with Fréchet distribution and conventional CDT model on S/4 measurement top points

**Fig. 19:**
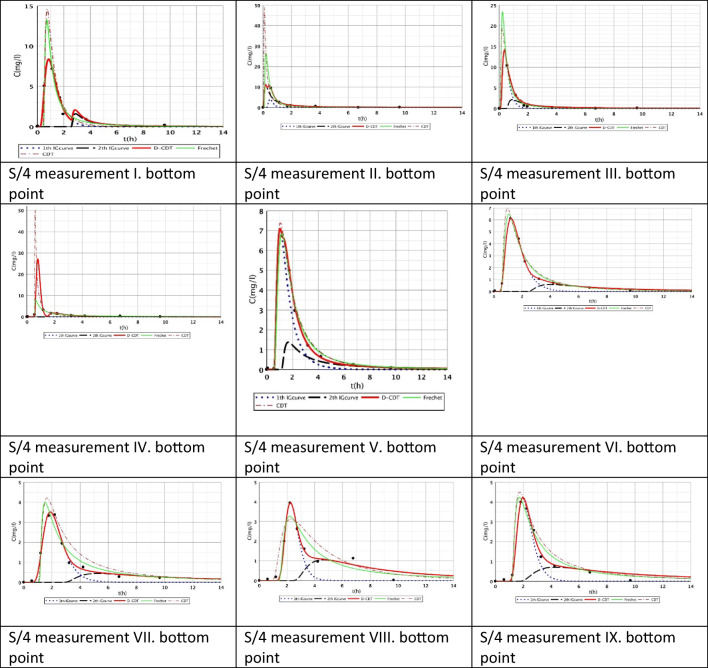
Fitting results of D-CDT model compared with Fréchet distribution and conventional CDT model on S/4 measurement bottom points

## Data Availability

All data generated or analyzed during this study are included in this published article (and its supplementary information files).

## Appendix 1

**Table 7:** Main data of own tracer measurement

Reference number of measurement	S/1	S/2	S/3	S/4
Source	Own measurements			
Date	09.02.2007	10.07.2007	02.08.2008	05.29.2008
Type of tracer	LiCl			
Amount of tracer (g)	8–17			
Geographical location	Hódmezővásárhely, Hungary			
Source of wastewater	milk room			
Medium	4–12-mm gravel			
Plant	Sedge			
Distribution and collection pipe	DN 125 PVC pipe perforated with 4-mm holes			
Inlet and outlet system	DN 160 PVC pipe			
Age of wetland	1 week	1.5 months	5 months	8 months
Dimensions of media body	6.85 × 3.7 × 0.6			
Evaporation at measurement (mm/d)	Negligible			
Porosity (-)	0.27	0.23	0.17	0.17
Hydraulic loading rate (m^3^/d)	29.4	31.1	16.93	14.34
Area under C-t function at 100% tracer response (h * mg/l)	6.669	6.305	11.582	13.674
Area of C-t function at 100% tracer response, corrected for evaporation (h * mg/l)	6.669	6.305	11.582	13.674
Distance between the distribution and collection pipes (m)	0,47; 2,21; 3,87	0,47; 2,21; 3,87	0,47; 2,21; 3,87	0,47; 2,21; 3,87
Porous velocity (m/d)	83.76	104.01	76.61	64.89
Parameter *b* = *L*/*v*_x_ (d)	0.06	0.04	0.06	0.07
Parameter *b* = *L*/*v*_x_ (h)	1.33	1.07	1.46	1.72

**Table 8:** Data series used for statistical research, 09.02.2007

S/1	Area under function: 6,6694 [h*mg/l]											
I.t	time [h]	0,00	0,25	0,83	1,75	2,80	3,78	4,75	6,75	8,75	10,67	
	C [mg/l]	0,00	14,39	3,37	0,66	0,19	0,02	0,01	0,00	0,00	0,00	
I.b	time [h]	0	0,25	0,8	1,8	2,8	3,8	4,8	6,8	8,8	10,7	
	C [mg/l]	0	14,4	2,4	1	0,3	0	0,1	0	0	0	
II.t	time [h]	0	0,25	0,9	1,8	2,8	3,8	4,8	6,8	8,8	10,7	
	C [mg/l]	0	11	2,2	0,5	0,1	0	0	0	0	0	
II.b	time [h]	0	0,25	0,9	1,8	2,8	3,8	4,8	6,8	8,8	10,7	
	C [mg/l]	0	11	3,6	0,4	0,1	0	0	0	0	0	
III.t	time [h]	0	0,25	0,9	1,8	2,9	3,8	4,8	6,8	8,8	10,7	
	C [mg/l]	0	28,7	3,3	0,2	0	0	0	0	0	0	
III.b	time [h]	0	0,25	0,9	1,8	2,9	3,8	4,8	6,8	8,8	10,7	
	C [mg/l]	0	28,7	4,1	0,2	0	0	0	0	0	0,04	
IV.t	time [h]	0	0,93	1,8	3,8	4,8	6,8	8,8	11			
	C [mg/l]	0	3,5	2,7	0,3	0,1	0,1	0	0			
IV.b	time [h]	0	0,93	1,8	2,9	3,8	4,8	6,8	8,8	11		
	C [mg/l]	0	1,86	3	1,3	0,6	0,2	0,1	0	0		
V.t	time [h]	0	0,97	1,8	3,9	4,8	6,8	8,8	11			
	C [mg/l]	0	0,39	3,3	0,4	0,2	0,1	0	0			
V.b	time [h]	0	0,97	1,8	3,9	4,8	6,8	8,8	11			
	C [mg/l]	0	0,3	3,6	0,6	0,2	0,1	0	0			
VI.t	time [h]	0	0,98	1,8	3,9	4,8	6,9	8,9	11			
	C [mg/l]	0	0,71	4,2	0,7	0,2	0,1	0	0			
VI.b	time [h]	0	0,98	1,8	3,9	4,8	6,9	8,9	11			
	C [mg/l]	0	0,64	4,3	0,9	0,4	0,1	0	0			
VII.t	time [h]	0	0,7	1,3	1,8	2,4	2,8	3,2	4,1	5,1	7,13	9,1
	C [mg/l]	0	0,1	5,3	2,9	0,6	0,3	0,2	0,1	0,1	0,05	0,02
VII.b	time [h]	0	0,57	1,1	1,6	2,1	2,5	3,1	3,6	4,6	9,1	
	C [mg/l]	0	0,05	2,3	3,8	1,6	0,6	0,5	0,4	0,2	0,05	
VIII.t	time [h]	0	0,58	0,7	1,1	1,3	1,6	1,9	2,1	2,4	2,55	2,81
		3,05	3,25	3,6	4,1	4,6	5,1	7,2	9,1	11		
	C [mg/l]	0	0,05	0,1	0,3	2,6	3,6	2,9	2,2	1,2	1	0,65
		0,47	0,44	0,4	0,2	0,2	0,1	0,1	0,1	0		
VIII.b	time [h]	0	0,58	0,7	1,1	1,3	1,6	1,9	2,1	2,4	2,57	2,8
		3,0667	3,25	3,6	4,6	5,1	6,1	7,2	9,1	11		
	C [mg/l]	0	0,05	0,1	0,7	2,8	4,3	3,3	2,3	1,4	1,17	0,81
		0,52	0,58	0,4	0,3	0,2	0,3	0,1	0,1	0		
IX.t	time [h]	0	0,73	1,4	1,9	2,4	2,8	3,3	5,1	6,2	7,18	11,15
	C [mg/l]	0	0,06	7,6	3,4	1,2	0,7	0,5	0,1	0,2	0,08	0,04
IX.b	time [h]	0	0,6	1,1	1,6	2,1	2,6	3,1	3,6	4,6	9,15	
	C [mg/l]	0	0,05	2	6,1	2,6	1,1	0,6	0,4	0,2	0,06	

**Table 9:** Data series used for statistical research, 10.07.2007

S/2	Area under function: 6305 [h*mg/l]											
I.t	time [h]	0	0,2	0,8	1,3	2,3	4,6	6,6	11			
	C [mg/l]	0	10,8	1	0,8	0,2	0	0	0			
I.b	time [h]	0	0,05	0,5	1	1,9	3,6	8,6				
	C [mg/l]	0	0,05	8,1	3,6	0,6	0,1	0				
II.t	time [h]	0	0,07	0,2	0,5	0,8	1,1	1,3	1,9	2,3	3,58	4,58
		6,5667	8,58	11								
	C [mg/l]	0	0,19	7,2	5,3	2,2	0,9	0,5	0,4	0,1	0,1	0,07
		0,02	0,02	0								
II.b	time [h]	0	0,07	0,2	0,5	0,8	1,1	1,3	1,9	2,3	3,6	4,58
		6,6167	8,58									
	C [mg/l]	0	0,04	4,1	5,3	3,6	2,6	1,2	0,5	0,4	0,26	0,13
		0,02	0,01									
III.t	time [h]	0	0,23	0,8	1,3	2,4	4,6	6,7	11			
	C [mg/l]	0	25,8	2,7	0,2	0	0	0	0			
III.b	time [h]	0	0,08	0,5	1,1	1,9	3,6	8,6				
	C [mg/l]	0	7,73	5,7	0,5	0,2	0	0				
IV.t	time [h]	0	0,23	0,9	1,4	1,9	2,6	3,6	4,6	6,7	10,6	
	C [mg/l]	0	2,44	3,1	0,8	0,5	0,4	0,1	0	0	0,01	
IV.b	time [h]	0	0,1	0,5	1,1	1,6	2,1	3,1	4,1	8,6		
	C [mg/l]	0	0,8	2	2,1	1,8	0,7	0,2	0,1	0		
V.t	time [h]	0	0,12	0,3	0,6	0,9	1,1	1,4	1,6	1,9	2,08	2,58
		3,1	3,65	4,1	4,6	6,7	8,6	11				
	C [mg/l]	0	0,05	0,1	4,7	3,5	2,1	1,2	0,8	0,5	0,45	0,28
		0,2	0,16	0,1	0,1	0	0	0				
V.b	time [h]	0	0,12	0,3	0,6	0,9	1,1	1,4	1,6	1,9	2,08	2,6
		3,0833	3,65	4,1	4,6	6,7	8,6	11				
	C [mg/l]	0	0,13	0,4	4,5	3,6	2,1	1,2	0,9	0,5	0,49	0,24
		0,33	0,16	0,1	0,1	0	0	0				
VI.t	time [h]	0	0,27	0,9	1,4	1,9	2,6	3,7	4,7	6,7	10,6	
	C [mg/l]	0	0,62	4	1	0,3	0,2	0,1	0,1	0	0,02	
VI.b	time [h]	0	0,13	0,6	1,1	1,6	2,1	3,1	4,1	8,7		
	C [mg/l]	0	0,04	8,1	2	0,6	0,3	0,2	0,1	0,1		
VII.t	time [h]	0	0,27	0,9	1,4	1,9	2,4	2,8	3,7	4,7	6,7	8,66
	C [mg/l]	0	0,1	5,3	2,9	0,6	0,3	0,2	0,1	0,1	0,05	0,02
VII.b	time [h]	0	0,13	0,6	1,1	1,6	2,1	2,6	3,1	4,1	8,67	
	C [mg/l]	0	0,05	2,3	3,8	1,6	0,6	0,5	0,4	0,2	0,05	
VIII.t	time [h]	0	0,15	0,3	0,6	0,9	1,2	1,4	1,6	1,9	2,12	2,38
		2,6167	2,82	3,1	3,7	4,1	4,7	6,7	8,7	11		
	C [mg/l]	0	0,05	0,1	0,3	2,6	3,6	2,9	2,2	1,2	1	0,65
		0,47	0,44	0,4	0,2	0,2	0,1	0,1	0,1	0		
VIII.b	time [h]	0	0,15	0,3	0,6	0,9	1,1	1,4	1,7	1,9	2,13	2,36
		2,6333	2,82	3,1	4,1	4,7	5,7	6,7	8,7	11		
	C [mg/l]	0	0,05	0,1	0,7	2,8	4,3	3,3	2,3	1,4	1,17	0,81
		0,52	0,58	0,4	0,3	0,2	0,3	0,1	0,1	0		
IX.t	time [h]	0	0,3	0,9	1,4	2	2,4	2,8	4,7	5,7	6,75	10,71
	C [mg/l]	0	0,06	7,6	3,4	1,2	0,7	0,5	0,1	0,2	0,08	0,04
IX.b	time [h]	0	0,17	0,7	1,2	1,7	2,2	2,6	3,2	4,2	8,72	
	C [mg/l]	0	0,05	2	6,1	2,6	1,1	0,6	0,4	0,2	0,06	

**Table 10:** Data series used for statistical research, 02.08.2008

S/3	Area under function: 11,582 [h*mg/l]											
I.t	time [h]	0	0,05	0,7	1,2	2,3	4,5	6,5				
	C [mg/l]	0	0,01	7,6	5,3	2,6	0,9	0,6				
I.b	time [h]	0	0,05	0,5	1	1,7	3,2	5,5				
	C [mg/l]	0	0,01	0,5	3,5	4,6	2,8	1,3				
II.t	time [h]	0	0,05	0,7	1,2	2,3	4,5	6,5				
	C [mg/l]	0	0,01	4,2	5,6	2,1	0,8	0,5				
II.b	time [h]	0	0,07	0,5	1	1,7	3,2	5,5				
	C [mg/l]	0	0,02	0,5	2,2	4,1	3,3	1,2				
III.t	time [h]	0	0,07	0,7	1,3	2,3	4,5	6,5				
	C [mg/l]	0	0,01	5,1	5,9	2,1	1	0,5				
III.b	time [h]	0	0,08	0,5	1	1,7	3,2	5,5				
	C [mg/l]	0	0,03	10	12	5,6	2,1	1				
IV.t	time [h]	0	0,08	0,8	1,3	1,8	2,3	2,5	3,2	6,5		
	C [mg/l]	0	0,01	0,7	4,3	5	1,3	3,7	2,7	0,8		
IV.b	time [h]	0	0,1	0,5	1	1,5	2	3	3,9	5,5		
	C [mg/l]	0	0,04	1,6	1,1	0,5	1,9	3,3	2,3	2		
V.t	time [h]	0	0,08	0,8	1,3	1,8	2,3	2,5	3,3	6,5		
	C [mg/l]	0	0,01	0,1	2	4,1	1,5	3,6	2,7	0,8		
V.b	time [h]	0	0,1	0,5	1	1,5	2	3	4	5,5		
	C [mg/l]	0	0,01	0,3	0,5	3,4	4,6	3,4	2,3	1,2		
VI.t	time [h]	0	0,1	0,8	1,3	1,8	2,3	2,5	3,3	6,5		
	C [mg/l]	0	0,01	0,1	2,1	4,4	1,6	4,1	2,8	0,8		
VI.b	time [h]	0	0,12	0,5	1	1,5	2	3	4	5,5		
	C [mg/l]	0	0,01	0,1	0,9	5,4	5,7	3,9	2,8	1,5		
VII.t	time [h]	0	0,8	1,3	1,8	2,7	3,3	4,5	5,6	6,5		
	C [mg/l]	0	0,03	0,2	1,6	3,6	3,3	3,1	1,9	0,8		
VII.b	time [h]	0	0,55	1,1	1,5	2	2,5	3	4	5,5		
	C [mg/l]	0	0,02	0,1	0,8	1,5	3,6	2,8	2,5	1,6		
VIII.t	time [h]	0	0,82	1	1,1	2,3	2,7	3,3	4,6	6,6		
	C [mg/l]	0	0,01	0	0,4	1,6	2,6	3,1	2,1	0,9		
VIII.b	time [h]	0	0,55	1	1,5	2	2,5	3	4	5,6		
	C [mg/l]	0	0,02	0	0,1	0,4	2,1	1,6	2,3	1,9		
IX.t	time [h]	0	0,83	1,3	1,8	2,3	2,8	3,3	4,6	6,6		
	C [mg/l]	0	0,01	0	0,3	1,2	1,9	2,6	2,1	1,1		
IX.b	time [h]	0	0,57	1,1	1,5	2	2,6	3,1	4	5,6		

**Table 11:** Data series used for statistical research, 05.29.2008

S/4	Area under function: 13,674 [h * mg/l]											
I.t	time [h]	0	0,27	0,8	1,4	2,4	4,7	8,2				
	C [mg/l]	0	2,56	3,3	0,8	0,5	0,1	0,1				
I.b	time [h]	0	0,03	0,5	1,1	1,7	2	3,7	6,7	9,6		
	C [mg/l]	0	0,08	5,1	7,2	3,6	1,6	0,9	0,1	0,2		
II.t	time [h]	0	0,27	0,8	1,4	2,5	4,7	8,2				
	C [mg/l]	0	4,7	2,1	0,6	0,2	0,1	0				
II.b	time [h]	0	0,03	0,5	1,2	1,7	2	3,7	6,7	9,6		
	C [mg/l]	0	0,27	9,5	2,6	0,7	0,9	0,6	0,1	0,2		
III.t	time [h]	0	0,28	0,8	1,5	2,5	4,7	8,2				
	C [mg/l]	0	0,58	2	1,1	0,3	0,1	0				
III.b	time [h]	0	0,05	0,5	1,2	1,7	2	3,7	6,7	9,6		
	C [mg/l]	0	0,02	10	3,2	0,7	0,5	0,2	0	0,1		
IV.t	time [h]	0	0,3	0,8	1,5	2	2,7	3,7	4,7	8,2		
	C [mg/l]	0	0,12	1,8	2	1,5	0,7	0,3	0,1	0,1		
IV.b	time [h]	0	0,07	0,5	1,2	1,8	2,2	3,2	4,2	6,7	9,62	
	C [mg/l]	0	0,07	1	3,3	1,6	1,6	0,7	0,4	0,4	0,09	
V.t	time [h]	0	0,32	0,8	1,5	2	2,7	3,7	4,7	8,2		
	C [mg/l]	0	0,04	0,9	4,7	3,9	1,7	0,7	0,4	0,1		
V.b	time [h]	0	0,07	0,6	1,2	1,8	2,2	3,2	4,2	6,7	9,63	
	C [mg/l]	0	0,06	0,4	6,7	5	3	1,2	0,7	0,2	0,08	
VI.t	time [h]	0	0,32	0,8	1,5	2	2,7	3,8	4,8	8,2		
	C [mg/l]	0	0,04	3,8	4,1	3,5	1,3	0,5	0,3	0,1		
VI.b	time [h]	0	0,08	0,6	1,2	1,8	2,2	3,2	4,2	6,8	9,65	
	C [mg/l]	0	0,07	0,7	6,1	4,4	2,5	1,1	0,7	0,3	0,08	
VII.t	time [h]	0	0,83	1,5	2	2,5	2,9	3,8	4,8	8,3		
	C [mg/l]	0	0,19	2,2	3	1,6	1,1	0,5	0,3	0,1		
VII.b	time [h]	0	0,58	1,2	1,8	2,2	2,7	3,2	4,2	6,8	9,65	
	C [mg/l]	0	0,07	1,5	3,3	3,4	1,9	1	0,8	0,3	0,23	
VIII.t	time [h]	0	0,87	1,5	2	2,5	3	3,8	4,8	8,3		
	C [mg/l]	0	0,05	0,9	2,8	1,8	1,7	0,9	0,5	0,2		
VIII.b	time [h]	0	0,6	1,2	1,8	2,2	2,7	3,3	4,2	6,8	9,68	
	C [mg/l]	0	0,07	0,2	2	4	2,6	1,6	1	1,1	0,02	
IX.t	time [h]	0	0,87	1,5	2	2,5	3	3,8	4,8	8,3		
	C [mg/l]	0	0,04	0,9	3,4	2,7	1,2	0,8	0,5	0,2		
IX.b	time [h]	0	0,62	1,2	1,8	2,3	2,8	3,3	4,3	6,8	9,68	
	C [mg/l]	0	0,08	0,3	4	3,7	2,6	1,2	0,8	0,5	0,04	

## Appendix 2

Tables 12, 13, 14, 15, 16, 17, 18, and 19 summarize the S/1, S/2, S/3, and S/4 top and bottom measurement results and the fitting parameters of the model.

**Table 12:** The S/1 top points fitting parameters of D-CDT model

Ref. number	Measurement data			Fitting parameters of 1st and 2nd inverse Gaussian distribution curves							
	Area of response curve (h * mg/l)	*L* (m)	*b*_1_ = *L*/*v*_x_ (h)	*s*	*a*_1_	*b*_1_	*c*_1_	*a*_2_	*b*_2_	*c*_2_	*R*^2^
I	6,67	0,47	0,13	0,40	1,48	0,22	0,05	0,80	0,79	0,29	0,999988
II	6,67	0,47	0,13	0,65	1,05	0,39	0,02	0,97	0,45	0,78	0,999976
III	6,67	0,47	0,13	0,66	1,92	0,20	0,10	0,21	0,36	0,66	1,000000
IV	6,67	2,21	0,63	0,60	2,22	1,12	0,37	1,47	1,57	0,94	0,999842
V	6,67	2,21	0,63	0,80	19,10	2,19	0,02	12,65	13,25	0,92	0,999673
VI	6,67	2,21	0,63	0,75	30,60	1,65	0,02	15,43	2,05	1,49	0,999701
VII	6,67	3,87	1,11	0,65	15,63	1,26	0,18	4,45	8,22	0,53	0,987500
VIII	6,67	3,87	1,11	0,48	6,72	1,07	0,71	5,85	11,71	0,92	0,993271
IX	6,67	3,87	1,11	0,60	36,32	1,13	0,19	0,55	1,50	1,71	0,999400

**Table 13:** The S/1 bottom points fitting parameters of D-CDT model

Ref. number	Measurement data			Fitting parameters of 1st and 2nd inverse Gaussian distribution curves							
	Area of response curve (h * mg/l)	*L* (m)	*b*_1_ = *L*/*v*_x_ (h)	*s*	*a*_1_	*b*_1_	*c*_1_	*a*_2_	*b*_2_	*c*_2_	*R*^2^
I	6,67	0,47	0,13	0,55	0,90	0,21	0,04	0,76	0,95	0,71	0,999949
II	6,67	0,47	0,13	0,65	1,05	0,39	0,02	0,37	0,57	0,65	0,999999
III	6,67	0,47	0,13	0,63	3,38	0,20	0,03	2,10	0,59	0,42	0,999998
IV	6,67	2,21	0,63	0,80	5,97	1,46	0,35	2,26	1,87	2,45	0,999796
V	6,67	2,21	0,63	0,67	22,76	2,14	0,01	2,08	5,72	2,68	0,999984
VI	6,67	2,21	0,63	0,75	29,74	1,72	0,01	2,12	1,72	3,01	0,999981
VII	6,67	3,87	1,11	0,60	17,19	1,45	0,11	5,36	11,39	1,26	0,997683
VIII	6,67	3,87	1,11	0,57	21,15	1,48	0,27	2,82	10,51	1,76	0,996894
IX	6,67	3,87	1,11	0,80	22,18	1,42	0,20	5,43	1,79	1,33	0,999408

**Table 14:** The S/2 top points fitting parameters of D-CDT model

Ref. number	Measurement data			Fitting parameters of 1st and 2nd inverse Gaussian distribution curves							
	Area of response curve (h * mg/l)	*L* (m)	*b*_1_ = *L*/*v*_x_ (h)	*s*	*a*_1_	*b*_1_	*c*_1_	*a*_2_	*b*_2_	*c*_2_	*R*^2^
I	6,31	0,47	0,11	0,57	1,70	0,29	0,08	0,09	5,41	0,20	0,995905
II	6,31	0,47	0,11	0,65	0,49	0,67	0,04	0,47	6,65	0,21	0,975735
III	6,31	0,47	0,11	0,77	1,65	0,22	0,02	0,30	0,23	0,59	0,999996
IV	6,31	2,21	0,51	0,70	1,65	0,68	0,05	1,42	11,59	1,70	0,987438
V	6,31	2,21	0,51	0,44	3,45	0,66	0,08	2,72	9,52	0,32	0,991427
VI	6,31	2,21	0,51	0,67	3,75	0,58	0,12	3,35	51,41	0,54	0,997636
VII	6,31	3,87	0,89	0,70	6,37	0,76	0,02	5,73	0,92	0,59	0,995639
VIII	6,31	3,87	0,89	0,49	8,04	1,09	0,23	5,47	9,77	0,50	0,992866
IX	6,31	3,87	0,89	0,60	5,56	0,66	0,37	3,48	1,16	0,70	0,998058

**Table 15:** The S/2 bottom points fitting parameters of D-CDT model

Ref. number	Measurement data			Fitting parameters of 1st and 2nd inverse Gaussian distribution curves							
	Area of response curve (h * mg/l)	*L* (m)	*b*_1_ = *L*/*v*_x_ (h)	*s*	*a*_1_	*b*_1_	*c*_1_	*a*_2_	*b*_2_	*c*_2_	*R*^2^
I	6,31	0,47	0,11	0,80	2,17	0,64	0,10	0,89	1,72	0,80	0,999939
II	6,31	0,47	0,11	0,70	1,54	0,68	0,00	1,29	6,47	0,49	0,996188
III	6,31	0,47	0,11	0,63	0,39	0,50	0,00	0,28	0,43	0,09	0,921434
IV	6,31	2,21	0,51	0,44	2,47	0,98	0,00	2,31	1042,87	0,00	0,861043
V	6,31	2,21	0,51	0,34	3,06	0,46	0,09	0,68	29,98	0,52	0,995669
VI	6,31	2,21	0,51	0,63	3,05	0,55	0,14	1,65	4,71	0,47	0,999236
VII	6,31	3,87	0,89	0,61	4,76	0,91	0,20	2,68	26,57	0,93	0,997206
VIII	6,31	3,87	0,89	0,64	11,18	1,30	0,07	4,33	6,79	1,24	0,995287
IX	6,31	3,87	0,89	0,67	18,65	1,05	0,01	4,97	1,15	0,77	0,989086

**Table 16:** The S/3 top points fitting parameters of D-CDT model

Ref. number	Measurement data			Fitting parameters of 1st and 2nd inverse Gaussian distribution curves							
	Area of response curve (h * mg/l)	*L* (m)	*b*_1_ = *L*/*v*_x_ (h)	*s*	*a*_1_	*b*_1_	*c*_1_	*a*_2_	*b*_2_	*c*_2_	*R*^2^
I	11,58	0,47	0,15	0,75	2,93	1,09	0,17	1,85	2,28	1,83	0,990438
II	11,58	0,47	0,15	0,43	9,37	0,90	0,19	4,37	3,11	0,80	0,995902
III	11,58	0,47	0,15	0,53	9,91	0,93	0,15	3,05	2,47	1,63	0,996642
IV	11,58	2,21	0,69	0,47	13,16	1,16	0,49	1,60	1,78	2,18	0,975159
V	11,58	2,21	0,69	0,31	14,26	1,13	0,58	1,81	2,66	0,12	0,994278
VI	11,58	2,21	0,69	0,32	15,16	1,06	0,61	1,60	2,26	2,14	0,992411
VII	11,58	3,87	1,22	0,70	29,61	3,11	0,01	9,32	1,70	3,51	0,972126
VIII	11,58	3,87	1,22	0,94	22,32	3,26	0,60	14,18	5,08	2,77	0,987436
IX	11,58	3,87	1,22	0,80	25,07	4,16	0,36	3,02	14,44	5,99	0,999000

**Table 17:** The S/3 bottom points fitting parameters of D-CDT model

Ref. number	Measurement data			Fitting parameters of 1st and 2nd inverse Gaussian distribution curves							
	Area of response curve (h * mg/l)	*L* (m)	*b*_1_ = *L*/*v*_x_ (h)	*s*	*a*_1_	*b*_1_	*c*_1_	*a*_2_	*b*_2_	*c*_2_	*R*^2^
I	11,58	0,47	0,15	0,73	7,14	0,89	0,06	0,42	2,97	1,37	0,989761
II	11,58	0,47	0,15	0,46	13,47	1,32	0,24	2,03	1,88	2,77	0,969585
III	11,58	0,47	0,15	0,92	3,34	0,71	0,17	0,35	0,19	1,64	0,943776
IV	11,58	2,21	0,69	0,10	4,42	0,69	0,11	3,16	2,96	1,54	0,833379
V	11,58	2,21	0,69	0,52	19,61	1,86	0,20	4,79	1,98	2,20	0,974708
VI	11,58	2,21	0,69	0,52	26,44	0,28	0,55	5,84	1,43	2,24	0,932704
VII	11,58	3,87	1,22	0,51	25,07	2,41	0,57	5,19	2,14	3,31	0,965969
VIII	11,58	3,87	1,22	0,27	26,88	2,24	0,78	8,45	3,53	2,69	0,976594
IX	11,58	3,87	1,22	0,85	32,07	4,61	0,09	22,10	4,06	2,52	0,991358

**Table 18:** The S/4 top points fitting parameters of D-CDT model

Ref, number	Measurement data			Fitting parameters of 1st and 2nd inverse Gaussian distribution curves							
	Area of response curve (h * mg/l)	*L* (m)	*b*_1_ = *L*/*v*_x_ (h)	*s*	*a*_1_	*b*_1_	*c*_1_	*a*_2_	*b*_2_	*c*_2_	*R*^2^
I	13,67	0,47	0,17	0,40	5,86	0,52	0,01	4,68	61,58	0,37	0,9639
II	13,67	0,47	0,17	0,20	0,51	0,48	0,00	0,00	0,29	0,17	0,9790
III	13,67	0,47	0,17	0,75	3,48	0,33	0,16	0,72	198,95	0,73	0,9895
IV	13,67	2,21	0,82	0,72	6,90	0,54	0,54	1,04	71,94	1,41	0,9885
V	13,67	2,21	0,82	0,48	6,92	1,50	0,29	6,81	15,79	0,68	0,9887
VI	13,67	2,21	0,82	0,42	7,92	1,27	0,11	2,16	18,12	1,27	0,9813
VII	13,67	3,87	1,43	0,33	12,25	1,87	0,33	10,10	520,97	2,25	0,9732
VIII	13,67	3,87	1,43	0,30	12,16	1,88	0,59	9,28	54,03	1,80	0,9547
IX	13,67	3,87	1,43	0,29	13,57	1,57	0,75	10,59	64,51	1,11	0,9930

**Table 19:** The S/4 bottom points fitting parameters of D-CDT model

Ref. number	Measurement data			Fitting parameters of 1st and 2nd inverse Gaussian distribution curves							
	Area of response curve (h * mg/l)	*L* (m)	*b*_1_ = *L*/*v*_x_ (h)	*s*	*a*_1_	*b*_1_	*c*_1_	*a*_2_	*b*_2_	*c*_2_	*R*^2^
I	13,67	0,47	0,17	0,74	4,47	1,24	0,02	1,15	9,65	2,50	0,989527
II	13,67	0,47	0,17	0,12	2,45	0,46	0,15	0,53	7,63	0,00	0,991407
III	13,67	0,47	0,17	0,56	1,91	0,55	0,00	1,39	21,05	0,44	0,990961
IV	13,67	2,21	0,82	0,70	6,22	0,53	0,32	1,67	3,42	1,37	0,993795
V	13,67	2,21	0,82	0,65	2,18	1,21	0,46	1,89	11,20	1,04	0,999333
VI	13,67	2,21	0,82	0,65	5,95	1,52	0,17	5,80	12,16	2,09	0,998708
VII	13,67	3,87	1,43	0,45	14,37	2,14	0,17	8,38	133,85	2,47	0,989126
VIII	13,67	3,87	1,43	0,35	17,63	1,72	0,76	8,27	9,46	2,09	0,974937
IX	13,67	3,87	1,43	0,41	11,83	1,66	0,61	9,41	14,36	1,31	0,989607

## Nomenclature

CW: Constructed wetland
FSCW: Free-surface flow constructed wetland
SFCW: Subsurface flow constructed wetland
VSFCW: Subsurface flow constructed wetland with vertical flow direction
HSFCW-C: Horizontal subsurface flow constructed wetland using coarse gravel filter media
HRT: Hydraulic retention time
D [m^2^/h]: Dispersion coefficient
D_x_ [m^2^/h]: Longitudinal dispersion coefficient
q [1/h]: Specific loading rate
x [m]: Longitudinal coordinate
CDT: Convection-dispersion tank
CSTR: Continuous stirred-tank reactor
LiCl: Lithium-chloride
C [mg/l]: Concentration
*v*_x_ [m/h]: Longitudinal velocity in porous regime
*L* [m]: Length of seepage zone
*R* [-]: Retention rate
*a*, *b*, *c*: Parameters of Inverse Gaussian distribution
S/1, S/2, S/3, and S/4: Reference numbers of own measurements
D-CDT: Divided convective-dispersive tank
*R*^2^: Statistical coefficient of determination
